# Biological Activity Evaluation of Some New Benzenesulphonamide Derivatives

**DOI:** 10.3389/fchem.2019.00634

**Published:** 2019-09-18

**Authors:** Florence Uchenna Eze, Uchechukwu Christopher Okoro, David Izuchukwu Ugwu, Sunday N. Okafor

**Affiliations:** ^1^Department of Pure and Industrial Chemistry, University of Nigeria, Nsukka, Nigeria; ^2^Department of Pharmaceutical and Medicinal Chemistry, University of Nigeria, Nsukka, Nigeria

**Keywords:** benzenesulphonamide, carboxamide, antioxidant, anti-inflammatory, antimicrobial

## Abstract

Bacterial resistance to antibiotics has become one of the most challenging problems of infectious disease treatment. Ten new derivatives of benzenesulphonamide bearing carboxamide functionality were synthesized and investigated for their *in vivo* anti-inflammatory, *in vitro* anti-microbial and anti-oxidant activities. The base promoted reactions of the appropriate amino acids with substituted benzenesulphonyl chlorides gave the benzene sulphonamides (**3a-j**) in excellent yields. Palladium mediated amidation of the benzenesulphonamides (**3a-j**) and butylamine gave the new carboxamides (**4a-j**) in excellent yield. Compounds **4a** and **4c** inhibited carrageenan induced rat-paw edema at 94.69, 89.66, and 87.83% each at 1, 2, and 3 h, respectively. In the antimicrobial activity, compound **4d** (MIC 6.72 mg/mL) was most potent against *E. coli*, compound **4h** (MIC 6.63 mg/mL) was the most active against *S. aureus*, compound **4a** (MIC 6.67 and 6.45 mg/mL) was most active against *P. aeruginosa* and *S. typhi*, respectively, compound **4f** (MIC 6.63 mg/mL) was the most active against *B. subtilis*, compounds **4e** and **4h** (MIC 6.63 mg/mL) each were the most active against *C. albicans*, while compound **4e** (MIC 6.28 mg/mL) was most active against *A. niger*. Only compound **4e** (IC_50_ 0.3287 mg/mL) had comparable activity with Vitamin C (IC_50_ 0.2090 mg/mL).

## Introduction

A major category of human diseases is bacterial infection. Resistance to almost all commercially available antibacterial drugs has been observed in both wild and laboratory strains of disease causing bacteria (Hayley and Paul, 2010). Prestinaci et al. (2015) reported four antibiotic-resistant pathogens of global concern, including *S. aureus, K. pneumoniae, S. typhi*, and *M. tuberculosis*. The resistance mechanisms are genetically encoded and under appropriate conditions, resistance genes can propagate through the environment (Christopher, 2000). This vast increase in resistance mechanisms often negates treatment by entire classes of antimicrobial compounds. Thus, the development of novel classes of antimicrobial compounds is urgently needed.

Reactive-Oxygen-Specie (ROS) is produced in an event of microbial invasion (Spooner and Yilmaz, 2011). Excess ROS can lead to oxidative stress (Circu and Aw, 2010). Some of these microorganisms are opportunistic pathogens implicated in chronic inflammatory conditions, including cystic fibrosis (Bylund et al., 2006).

Sulphonamides constitute an important class of drugs. They are quite stable and tolerated in human beings (Shet et al., 2013). They are the basis of several groups of drugs with various types of pharmacological agents possessing carbonic anhydrase inhibitory activity (Supuran, 2008), antibacterial (Ali et al., 2009), anticancer (Ghorab et al., 2014), anti-HIV (Selvam et al., 2008), antidiabetic (Hosseinzadeha et al., 2013), anti-influenza (Tang et al., 2011), antioxidant (Siddique et al., 2013), anti-inflammatory (Mahtab et al., 2014), antimicrobial (Chandak, 2012), antitrypanosomal (Papadopoulou et al., 2012), anticonvulsant (Bhat et al., 2006), anti-insomnia (Aissaoui et al., 2008), diuretics (Jainswal et al., 2004), and antileukemic (Nakayama et al., 2005) activities to mention but a few.

Carboxamides are ubiquitous in their function in drug molecules as a pharmacophore (Montalbetti and Falque, 2005). Carboxamides have been reported as antihelmintic (Ugwu et al., 2018a), antitubercular (Ugwu et al., 2014) anti-trypanosomal (Ugwu et al., 2018b) agents. They are also present in drug molecules used in the blockage of cholesterol synthesis (Graul and Castaner, 1997), treatment of hypertension and andina (Ananthanarayanan et al., 1993), blockade of angiotensin-II receptors (deGasparo and Whitebread, 1995), inhibition of angiotensin converting enzymes (Patchett, 1993), treatment of HIV (Roskoski, 2003), and management of heart disease (Hogan et al., 2000), to mention but a few.

This work was designed based on the reported biological activities of benzenesulphonamide derivatives and carboxamides and the need to develop newer antimicrobial agents that will have an added advantage of reducing the reactive oxygen species and inflammation implicated during microbial invasion while also acting as an antimicrobial agent using L-amino acids and substituted benzenesulphonamides.

We herein report the synthesis of some sulphonamides bearing carboxamide functionalities with good anti-inflammatory, antimicrobial, and comparable antioxidant activities.

## Experimental

### Synthesis of Substituted Benzene Sulphonamoylalkanamides (3a-j)

Sodium carbonate (NaCO_3_, 1.59 g, 15 mmol) was added to a solution of amino acids (**2**, 12.5 mmol) in water (15 mL) with continuous stirring until all the solutes dissolved. The solution was cooled to −5°C and the appropriate benzenesulphonyl chloride (**1**, 15 mmol) was added in four portions over a period of 1 h. The slurry was further stirred at room temperature for 4 h. The progress of the reaction was monitored using TLC (MeOH/DCM) 1:9). Upon completion, the mixture was acidified using 20% aqueous hydrochloric acid to pH2. The products (**3a-j**) were obtained in their analytical grade after washing with tartaric acid solution of pH 2.2. The products were dried over self-indicating fused silica gel in a desiccator (Ugwu et al., 2017).

Palladium catalyzed amidation of unactivated carboxylic acid and butylamine.

**Scheme 1:**
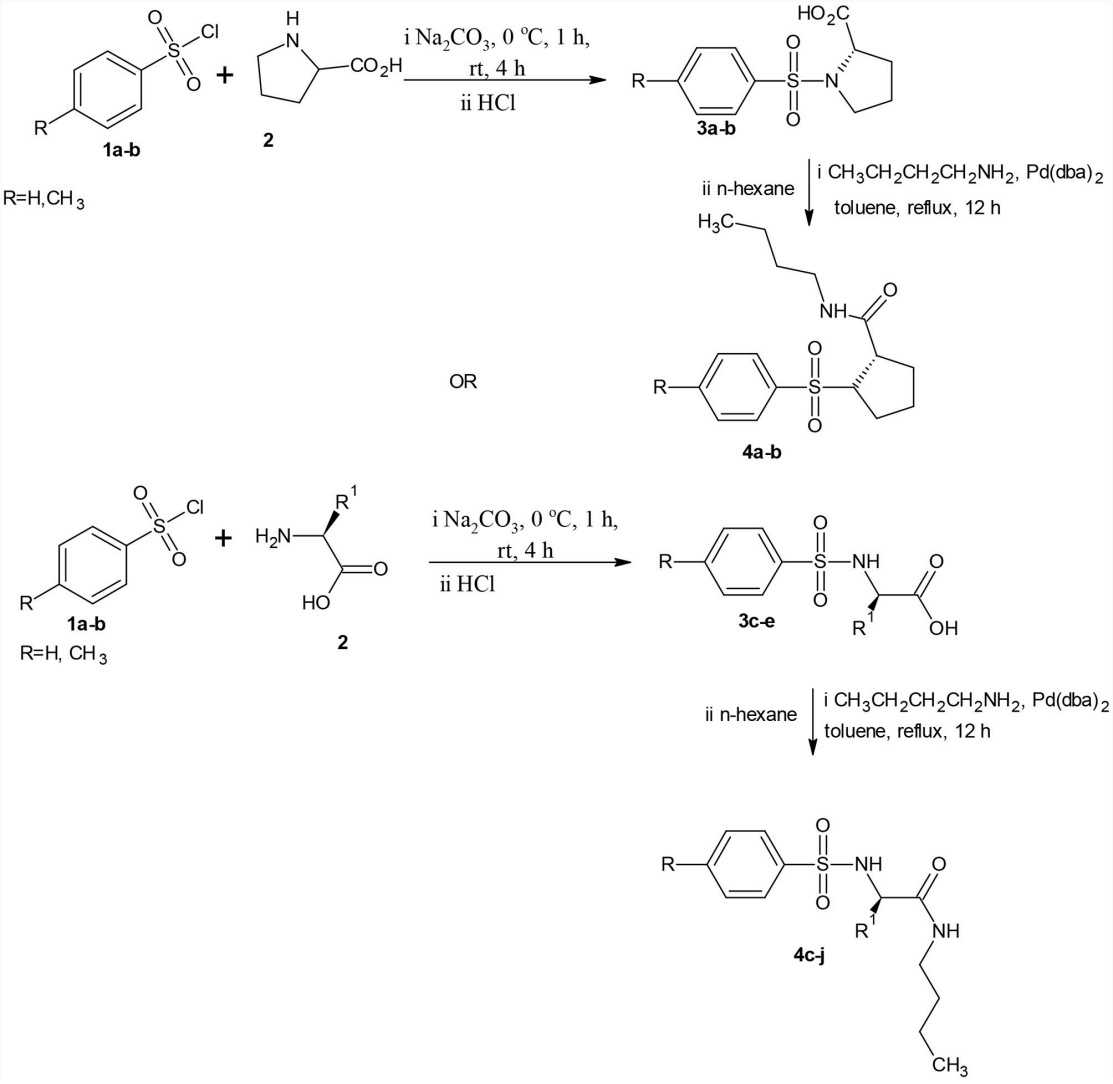
Synthetic route to the new carboxamides.

Butylamine (1.0 mmol) and Pd(dba)_2_ (0.1 mmol) at room temperature were added to a suspension of substituted benzenesulphonamides (**3a-j**, 1.0 mmol) in dry toluene (40 mL) equipped with Dean-Stark apparatus for azeotropic removal of water, and then refluxed for 12 h. On completion (as monitored by TLC) the amide products precipitated out in their pure form from the reaction mixture by adding 40 mL n-hexane. The carboxamides (**4a-j**) were obtained via suction filtration, washed with n-hexane and dried over fused silica gel or concentrated using rotary evaporator and dried over vacuum in the case of oily products (Scheme 1).

### *N*-Butyl-1-[(4-Methylphenyl)Sulphonyl]Pyrolidine-2-Carboxamide (4a)

Yield (0.22 g, 66.7%), Mp, 128–130°C. UV (λmax): 213.00 nm(ε = 427.5 m^2^/mol). FTIR (KBr, cm^−1^): 3,160 (NH), 2,959 (C-H aromatic), 2,871, 2,795 (C-H aliphatic), 1,629 (C = O), 1,569, 1,450 (C = C), 1,342, 1,394 (2S = O), 1,200, 1,156 (SO_2_NH), 1,092, 1,055 (C-N)0.1 HNMR (DMSOd_6_, 400 MHz)δ: 7.68–7.64 (m, 2H, ArH), 7.35–7.33 (m, 2H, ArH), 3.82 (m, 1H, CH-C = O), 3.22 (m, 1H, CHa of CH_2_-N), 3.09–3.07 (m, 1H, CHb of CH_2_-N), 2.67 (m, 2H, CH_2_-NH), 2.24 (s, 3H, CH_3_-Ar), 1.69 (m, 2H, CH_2_CHC = O), 1.47 (m, 2H, CH_2_-CH_2_-N), 1.28–1.26 (m, 4H, 2CH2, CH_2_CH_2_-CH_3_), 0.85–0.80 (m, 3H, CH_3_-CH_2_) 0.1^3^CNMR (DMSOd_6_, 400 MHz)δ: 175.22 (C = O), 143.17, 136.28, 130.07, 127.63 (four aromatic carbons), 63.71, 48.75, 38.99, 31.18, 30.29, 24.66, 21.50, 19.80,14.10 (nine aliphatic carbons). HRMS (m/z): 325.1588 (M+H), calculated, 325.1586.

### *N*-Butyl-1-(Phenylsulphonyl)Pyrrolidine-2-Carboxamide (4b)

Yield (0.27 g, 87.1%), Mp, 114–116°C. UV (λmax): 202.00 nm(ε = 398.3 m^2^/mol). FTIR (KBr, cm^−1^): 3,489 (NH), 2,960 (C-H aromatic), 2,874, 2,796 (C-H aliphatic), 1,629 (C = O), 1,569, 1,446 (C = C), 1,394, 1,334 (2S = O), 1,193, 1,156 (SO_2_NH), 1,088, 1,014 (C-N)0.1HNMR (DMSOd_6_, 400 MHz)δ: 7.80–7.78 (d, J = 8.24 Hz, 2H, ArH), 7.62–7.52 (m, 3H, ArH), 3.88–3.86 (t, J = 3.42 Hz, 1H, CH-C = O), 3.14–3.11 (t, J = 6.88 Hz, 2H, CH_2_-N), 2.68–2.65 (m, 2H, CH_2_-NH), 1.70 (m, 2H, CH_2_-CH-C = O), 1.51–1.43 (m, 2H, CH_2_-CH_2_-N), 1.29–1.24 (m, 4H, 2CH_2_, CH_2_-CH_2_-CH_3_), 0.84–0.81 (m, 3H, CH_3_-CH_2_)0.1^3^CNMR (DMSOd_6_, 400 MHz)δ: 174.99 (C = O), 139.20, 132.97, 129.61, 127.56 (four aromatic carbons), 63.74, 48.72, 31.18, 30.38, 24.67, 19.79, 14.11 (seven aliphatic carbons). HRMS (m/z): 311.1430 (M+H), calculated, 311.1429.

### *N*-Butyl-4-Methyl-2-(4-Methylbenzenesulphonamido)Pentanamide (4c)

Yield (0.31 g, 88.6%), Mp, 110–112°C. UV (λmax): 202.00 nm(ε = 445.2 m^2^/mol). FTIR (KBr, cm^−1^): 3,250 (NH), 3,049 (C-H aromatic), 2,960, 2,866, 2,751 (C-H aliphatic), 1,640 (C = O), 1,580, 1,461 (C = C), 1,394, 1,342 (2S = O), 1,286, 1,159 (SO_2_NH), 1,099, 977 (C-N)0.1HNMR (DMSOd_6_, 400 MHz)δ: 8.18–8.16 (d, J = 8.40 Hz, 1H, NH), 7.86–7.80 (m, 2H, Ar), 6.72–6.70 (m, 2H, ArH), 3.36–3.35 (t, J = 4.40 Hz, 1H, CH-C = O), 3.16–3.14 (m, 2H, CH_2_-NH), 3.06–3,01 (m, 6H, 3CH_2_), 2.46 (s, 3H, CH_3_-Ar), 1.14–1.10 (m, 9H, 3CH_3_). ^13^CNMR (DMSOd_6_, 400 MHz)δ: 167.86 (C = O), 154.70, 137.58, 131.70, 127.16, 125.18, 123.91 (six aromatic carbons), 64.26, 52.65, 46.07, 39.32, 36.45, 8.93 (six aliphatic carbons). HRMS (m/z): 340.1822 (M^+^), calculated, 340.1821.

### *N*-Butyl-4-Methyl-2-[(Phenylsulphonyl)Amino]Pentanamide (4d)

Yield (0.25 g, 78.1%), Mp, 116–118°C. UV (λmax): 202.00 nm(ε = 422.1 m^2^/mol). FTIR (KBr, cm^−1^): 3,258 (NH), 3,064 (C-H aromatic), 2,960, 2,870 (C-H aliphatic), 1,640 (C = O), 1,573, 1,428 (C = C), 1,387, 1,338 (2S = O), 1,290, 1,152 (SO_2_NH), 1,014, 973 (C-N)0.1HNMR (DMSOd_6_, 400 MHz)δ: 7.73–7.71 (d, J = 7.80 Hz, 2H, ArH), 7.56–7.47 (m, 3H, ArH), 3.19 (m, 1H, CH-C = O), 2.61–2.57 (m, 2H, CH_2_-NH), 1.72–1.65 (m, 1H, CH-(CH_3_)_2_), 1.44–1.38 (m, 2H, CH_2_-CHC = O), 1.31–1.18 (m, 4H, CH_2_), 0.91–0.87 (m, 3H, CH_3_), 0.82–0.72 (m, 6H, 2(CH_3_)). ^13^CNMR (DMSOd_6_, 400 MHz)δ: 174.30 (C = O), 141.38, 132.58, 129.37, 127.13 (four aromatic carbons), 56.48, 43.28, 29.77, 24.51, 23.47, 22.61, 19.72, 14.04 (eight aliphatic carbons). HRMS (m/z): 327.1744 (M+H), calculated, 327.1742.

### *N*-Butyl-2-{[(4-Methylphenyl)Sulphonyl]Amino}-4-(Methylsulphanyl)Butanamide (4e)

Yield (0.36 g, 100%), Mp, 124–126°C. UV (λmax): 202.00 nm(ε = 399.4 m^2^/mol). FTIR (KBr, cm^−1^): 3,250 (NH), 3,049 (C-H aromatic), 2,960, 2,922, 2,870 (C-H aliphatic), 2,624 (S-CH_3_), 1,640 (C = O), 1,580, 1,521, 1,435, 1,402 (C = C), 1,361, 1,316 (2S = O), 1,208, 1,152 (SO_2_NH), 1,088, 1,047 (C-N)0.1HNMR (DMSOd_6_, 400 MHz)δ: 7.62–7.60 (d, J = 7.32 Hz, 2H, ArH), 7.32–7.30 (d, J = 7.92 Hz, 2H, ArH), 3.17–3.14 (t, J = 5.18 Hz, 1H, CH-C = O), 2.67–2.64 (m, 2H, CH_2_-NH), 2.39–2.32 (m, 3H, CH_3_-Ar), 2.25 (s, 3H, CH_3_-S), 1.99–1.92 (t, J = 5.64 Hz, 2H, CH_2_-S), 1.78–1.76 (m, 2H, CH_2_-CH), 1.46–1.39 (m, 2H, CH_2_-CH_2_-NH), 1.29–1.20 (m, 2H, CH_2_-CH_3_), 0.87–0.84 (t, J = 5.20 Hz, 3H, CH_3_-CH_2_). ^13^CNMR (DMSOd_6_, 400 MHz)δ: 172.08 (C = O), 143.00, 137.84, 130.00, 127.28 (four aromatic carbons), 56.68, 41.18, 38.90, 33.54, 29.88, 21.48, 19.67, 15.03, 14.04 (nine aliphatic carbons). HRMS (m/z): 357.1310 (M-H), calculated, 357.1307.

### *N*-Butyl-2-(4-Methylphenylsulphonyl)-2-[(Phenylsulphonyl)Amino]Butanmide (4f)

Yield (0.30 g, 92%), Mp, 120–121°C. UV (λmax): 202.00 nm(ε = 406.9 m^2^/mol). FTIR (KBr, cm^−1^): 3,246 (NH), 3,067 (CH aromatic), 2,917, 2,918 (CH aliphatic), 2,657 (-S-CH_3_), 1,710 (C = O), 1,584, 1,416 (C = C aromatic),1,326, 1,233 (2S = O), 1,155, 1,088, 969, 928 (C-N)0.1HNMR (DMSOd_6_, 400 MHz)δ: 7.88–7.87 (d, J = 7.40 Hz, 2H, ArH), 7.65–7.64 (t, J = 7.32 Hz, 3H, ArH), 3.15–3.13 (t, J = 6.22 Hz7, 1H, CH-C = O), 2.66–2.64 (m, 2H, CH_2_-NH), 2.23 (s, 3H, CH_3_-S), 1.97–1.95 (m, 2H, CH_2_-S), 1.79–1.77 (m, 2H, CH_2_-CH), 1.42–1.38 (m, 2H, CH_2_-CH_2_-NH), 1.27–1.24 (m, 2H, CH_2_-CH_3_), 0.84–0.82 (t, J = 5.20 Hz, 3H, CH_3_-CH_2_). ^13^CNMR (DMSOd_6_, 400 MHz)δ: 173.38 (C = O), 143.46, 138.65, 130.50, 127.28 (four aromatic carbons), 56.68, 41.18, 38.90, 33.54, 29.88, 19.67, 15.03, 14.04 (eight aliphatic carbons). HRMS (m/z): 344.1229 (M^+^), calculated 344.1228.

### *N*-Butyl-3-Hydroxy-2-{[(4-Methyl)Sulphonyl]Amino}Butanamide (4g)

Yield (0.28 g, 84.8%), Mp, 162–164°C. UV (λmax): 213.00 nm(ε = 421.7 m^2^/mol). FTIR (KBr, cm^−1^): 3,399 (OH), 3,250 (NH), 2,959 (C-H aromatic), 2,934, 2,874 (C-H aliphatic), 1,603 (C = O), 1,513, 1,465 (C = C), 1,379, 1,327 (2S = O), 1,159, 1,122 (SO_2_NH), 1,092, 1,036 (C-N, C-O)0.1HNMR (DMSOd_6_, 400 MHz)δ:7.69–7.57 (m, 2H, ArH), 7.48–7.46 (m, 2H, ArH), 3.77–3.76 (d, J = 6,32, Hz, 1H, CH-C = O), 3.08–3.06 (m, 1H, CH-OH), 2.56–2.53 (m, 2H, CH_2_-NH), 1.41–1.39 (m, 2H, CH_2_-CH_2_-NH), 1.33–1.31 (m, 2H, CH_2_-CH_3_), 0.98–0.96 (d, J = 5.96 Hz, 3H, CH_3_-CHOH), 0.83–0.81 (m, 3H, CH_3_-CH_2_). ^13^CNMR (DMSOd_6_, 400 MHz)δ: 172.64 (C = O), 142.44, 132.94, 128.71, 127.84 (four aromatic carbons), 68.01, 60.14, 38.95, 29.70, 21.56, 19.64, 19.09, 14.02 (eight aliphatic carbons). HRMS (m/z): 327.1380 (M-H), calculated, 328.1379.

### *N*-Butyl-3-Hydroxy-2-[(Phenylsulphonyl)Amino]Butanamide (4h)

Yield (0.29 g, 90.6%), Mp, 140–142°C. UV (λmax): 213.00 nm(ε = 362.1 m^2^/mol). FTIR (KBr, cm^−1^): 3,401 (OH), 3,191 (NH), 3,056 (C-H aromatic), 2,960, 2,933, 2,870 (C-H aliphatic), 1,733 (C = O), 1,595, 1,528, 1,476 (C = C), 1,387, 1,305 (2S = O), 1,238, 1,141 (SO_2_NH), 1,077, 973 (C-N, C-O)0.1HNMR (DMSOd_6_, 400 MHz)δ: 7.75 (m, 2H, ArH), 7.59–7.51 (m, 3H, ArH), 3.70–3.69 (d, J = 4.56 Hz, 1H, CH-C = O), 3.082 (m, 1H, CH-OH), 2.67–2.65 (m, 2H, CH_2_-NH), 1.43–1.41 (m, 2H, CH_2_-CH_2_-NH), 1.28–1.22 (m, 2H, CH_2_-CH_3_), 0.92–0.89 (d, J = 5.96 Hz, 3H, CH_3_-CHOH), 0.84–0.79 (m, 3H, CH3-CH2). ^13^CNMR (DMSOd_6_, 400 MHz)δ: 171.60 (C = O), 140.44, 132.94, 129.61, 127.24 (four aromatic carbons), 68.01, 60.14, 38.95, 29.70, 19.64, 19.09, 14.02 (seven aliphatic carbons). HRMS (m/z): 315.1379 (M+H), calculated, 315.1378.

### *N*-Butyl-3-Hydroxy-2-{[(4-Methylphenyl)Sulphonyl]Amino}Propanamide (4i)

Yield (0.29 g, 90.6%), Mp = 134–136°C. UV (λmax): 203.00 nm(ε = 330.9 m^2^/mol). FTIR (KBr, cm^−1^): 3,466 (OH), 3,243 (NH), 3,060 (C-H aromatic), 2,960, 2,933, 2,873 (C-H aliphatic), 1,733 (C = O), 1,595, 1,494, 1,461 (C = C), 1,365, 1,324 (2S = O), 1,163, 1,122 (SO_2_NH), 1,092, 1,033 (C-N, C-O)0.1HNMR (DMSOd_6_, 400 MHz)δ: 7.89–7.87 (d, J = 7.36 Hz, 1H, NH), 7.62–7.61 (d, J = 6.44 Hz, 2H, ArH), 7.32–7.30 (d, J = 6.44 Hz, 2H, ArH), 3.51–3.48 (m, 1H, CH), 3.30 (s-br, 1H, OH), 2.47–2.46 (m, 2H, CH_2_-NH), 2.33(s, 3H, CH_3_-Ar), 1.62–1.58 (m, 2H, CH_2_-OH), 1.34–1.29 (m, 2H, CH_2_-CH_2_-NH), 1.09–1.01 (m, 2H, CH_2_-CH_3_) 0.76–0.71 (t, J = 5.48 Hz, 3H, CH_3_-CH_2_)0.1^3^CNMR (DMSOd_6_, 400 MHz)δ: 172.36 (C = O), 149.93, 147.24, 128.72, 124.78 (four aromatic carbons), 60.89, 37.32, 24.85, 21. 44, 19.54, 15.96, 11.46 (seven aliphatic carbons). HRMS (m/z): 314.1303 (M^+^), calculated, 314.1300.

### *N*-Butyl-3-Hydroxy-2-[(Phenylsulphonyl)Amino]Propanamide (4j)

Yield (0.28 g, 93.3%), Mp = 120–122°C. UV (λmax): 203.00 nm(ε = 364.7 m^2^/mol). FTIR (KBr, cm^−1^): 3,391 (OH), 3,243 (NH), 2,963 (C-H aromatic), 2,875 (C-H aliphatic), 1,733 (C = O), 1,599, 1,446 (C = C), 1,387, 1,320 (2S = O), 1,252, 1,160 (SO_2_NH), 1,092, 1,026 (C-N, C-O)0.1HNMR (DMSOd_6_, 400 MHz)δ: 7.94–7.93 (d, J = 7.36 Hz, 1H, NH), 7.63–7.62 (d, J = 6.44 Hz, 2H, ArH), 7.31–7.29 (d, J = 6.40 Hz, 3H, ArH), 3.53–3.49 (m, 1H, CH), 3.30 (s-br, 1H, OH), 2.44–2.42 (m, 2H, CH_2_-NH), 1.58–1.56 (m, 2H, CH_2_-OH), 1.44–1.42 (m, 2H, CH_2_-CH_2_-NH), 1.06–1.04 (m, 2H, CH_2_-CH_3_) 0.66–0.64 (t, J = 6.32 Hz, 3H, CH_3_-CH_2_)0.1^3^CNMR (DMSOd_6_, 400 MHz)δ: 170.76 (C = O), 148.43, 146.64, 127.74, 124.78 (four aromatic carbons), 63.44, 38.44, 37.32, 24.85, 15.96, 11.46 (six aliphatic carbons). HRMS (m/z): 299.1069 (M-H), calculated, 299.1066.

### *In silico* Studies

#### Drug Targets

Peroxisomes are essential organelles which participate in multiple important metabolic processes, including the β-oxidation of fatty acids, plasmalogen synthesis, and the metabolism of reactive oxygen species (ROS) (Islinger et al., 2012). Human peroxiredoxin 5 (PRDX5) (PDB code: 1HD2), aperoxisome, is a thioredoxin reductase which reduces H_2_O_2_, alkyl hydroperoxides and peroxynitrite (Knoops et al., 2011). PRDX5 is a novel type of mammalian thioredoxin peroxidase widely expressed in tissues and located cellularly to mitochondria, peroxisomes, and cytosol. Functionally, PRDX5 has been implicated in antioxidant protective mechanisms as well as in signal transduction in cells (Declercq et al., 2001).

Phosphodiesterase-4 (PDE4) (PDB code: 4WCU) is an enzyme found in some specific celltypes, and is involved in the degradation of the second messenger, cAMP. As a result, 4WCU has a pivotal role in cell signaling. This has made it a target for clinical drug development of various indications, including anti-inflammation and several others (Zhang et al., 2005).

Glucosamine-6-phosphate synthase (GlcN-6-P) (PDB code: 2VF5) is a very useful target in antimicrobial chemotherapy as outlined by Ezeokonkwo et al. (2017) and Festus et al. (2018). 2VF5 is responsible for the metabolism of hexosamine which is an important process in the biosynthesis of amino sugars. In the biosynthesis of amino sugars, uridine 5′-diphospho-*N*-acetyl-d-glucosamine (UDP-GlcNAc) is formed. UDP-GlcNAc an important component of thepeptido glycan layer mostly found in the bacterial and fungal cell walls. Inactivation of GlcN-6-P synthase for a short period is very dangerous for fungal cells.

#### Molecular Docking Studies

Three drugs targets were selected to study the *in silico* antioxidant, anti-inflammatory, and antibacterial activities of the synthesized compounds. The targets used for the antioxidant and anti-inflammatory studies are the human peroxiredoxin 5 (PDB code: 1HD2) and phosphodiesterase 4 (PDE4) (PDB: 4WCU), respectively. Glucosamine-6-phosphate synthase (PDB Code: 2VF5) was used for antibacteria study.

The 3-Dimensional crystal structures of 1HD2, 4WCU, and 2VF5 with their co-crystallized ligands were retrieved from the protein data bank repository (https://www.rcsb.org/). These proteins were treated in Discovery Studio where multiple chains and water of crystallization were removed. The synthesized compounds were drawn using Accelrys Draw 4.1. Both the prepared proteins and compounds were energy minimized using MMFF94x force field. The energy minimized compounds were docked into the binding cavities of the proteins. The binding free energy for each compound against the target was calculated. Biovia Discovery Studio v16.1.0.15350 software was used for analysis of molecular docking studies. Molinspiration software (www.molinspiration.com) was used to generate the physicochemical properties in Table 7.

### Biological Studies

#### *In vivo* Anti-inflammatory Activities Determination

Male albino rats weighing 300 g where purchased from the Department of Biochemistry, University of Nigeria, Nsukka, and kept at room temperature in a light controlled animal house. They were fasted with free access to water for at least 12 h prior to the experiments. The tested compounds were prepared as suspension in vehicle (0.5% methylcellulose) and celecoxib was used as a standard drug. The positive control received celecoxib while the negative control received only the vehicle. Oedema was produced by injecting 0.2 mL of a solution of 1% carrageenan in the hindpaw. The rats were injected intraperitoneally with 1 mL suspension in 0.5% methylcellulose of the tested compounds and reference drug. Paw volume was measured by water displacement with aplethysmometer (UGO BASILE) before, 0.5, 1, 2, and 3 h after treatment. The percentage was calculated by the following equation (Abdel-Aziz et al., 2014):

$$\begin{array}{l} \text{Anti}-\text{inflammatory activity}\left(\%\right)=\left(1-\text{D}/\text{C}\right)\times \;100 \end{array}$$

where D represents the difference in paw volume before and after drug administration to the rats and C represents the difference of volume in the control groups. The approval for the use of animal was obtained from the University of Nigeria committee on experimental animal use.

#### *In vitro* Antimicrobial Activity

The antimicrobial properties of the novel compounds were investigated by general sensitivity testing and minimum inhibitory concentration (MIC), with respect to freshly cultured targeted organisms. The seven organisms used in this study are two Gram positive bacteria [*Staphylococcus aureus* (ATCC 12600)*, Bacillus subtilis* (ATCC 6633)], three gram negative bacteria [*Eschericha coli (O157:H7), Pseudomonas aeruginosa* (ATCC 27853)*, Salmonellatyphi* (H56)], and two fungi [*Candida albicans* (ATCC MYA 2876)*, Aspergillus niger* (ATCC 16404)] were obtained from the Department of Pharmaceutics, University of Nigeria, Nsukka.

#### Antimicrobial Sensitivity Testing

Sensitivity test agar plates were seeded with 0.1 mL of overnight culture of microorganism. The seeded plates were allowed to set after which cups were made in each sector previously drawn on the backside of the bottom plate using marker. Using a sterile pipette, each cup was filled with six drops of their corresponding carboxamides (100 mg/mL). The solubility solvent was DMF. All the plates were incubated at 37°C for 24 h for bacteria and 48 h for fungi. Zones of clearance around each cup allowed inhibition and the diameter of such zones to be measured. The procedure was repeated for tetracycline (standard bacteria), fluconazole (fungi standard), and DMF (solvent). Muller Hinton agar was used for the fungi in place of nutrient agar for bacteria (Adeniyi and Odedola, 1996).

#### Minimum Inhibitory Concentration (MIC) Testing

Serial dilutions of the carboxamides were prepared from 100 mg/mL solution of the compounds to give 100, 50, 25, and 12.5 mg/mL. Six drops of each dilution was added to the corresponding cup of seeded microorganisms and the agar previously marked. The cork borer used to make the cup is 8 mm in diameter. The plates were incubated at 37°C for 24 and 48 h in the case of fungi. The diameter of the zone of inhibition was measured and the value subtracted from the diameter of the borer to give the inhibition zone diameter (IZD). The graph of IZD (Prestinaci et al., 2015) against the log of concentration was plotted for each plate containing a specific compound and a microorganism. The anti-log of the intercept on x-axis gives the MIC (Adeniyi and Odedola, 1996). The procedure was repeated for tetracycline and fluconazole.

### *In vitro* Antioxidant Studies

#### DPPH Radical Scavenging Activity

The new carboxamides were screened for free radical scavenging activity by 2,2-diphenyl-1-picrylhydrazyl (DPPH) method (Liyana-Pathiranan and Shahidi, 2005). Compounds of different concentrations were prepared in distilled ethanol, with 1 mL of each compound solutions having different concentrations (1.0, 2.0, 3.0, 4.0, and 5.0 mg/mL) were taken indifferent test tubes, 4 mL of 0.1 mM ethanol solution of DPPH was added and shaken vigorously. The test tubes were then incubated in a dark room temperature for 20 min. A DPPH blank was prepared without the compound and ethanol was used for the baseline correction. Changes (decrease) in the absorbance at 517 nm were measured using a UV-Visible spectrometer. The radical scavenging activities were expressed as the inhibition percentage and were calculated using:

$$\begin{array}{l} \text{DPPH radical scavenging activity}=\frac{\text{Ac }-\text{ As}}{\text{Ac}}*100, \end{array}$$

Where Ac, Absorbance of control; As, Absorbance of sample.

## Results and Discussion

### Chemistry

**Scheme 2:**
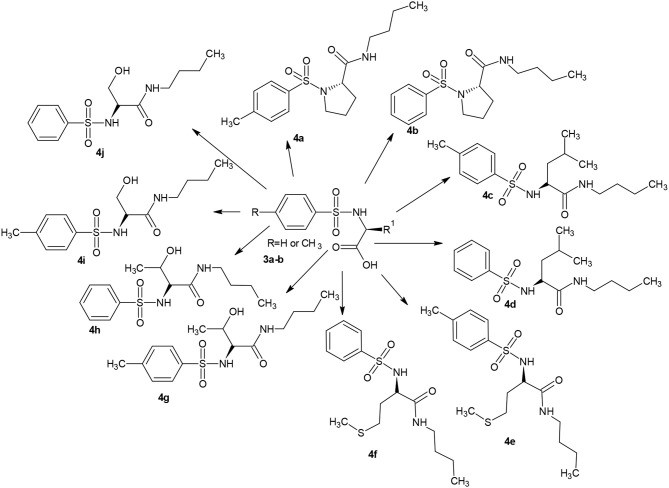
Butylamine derivatives of carboxamide.

Substituted benzenesulphonamides (**3a-j**) were synthesized from the reaction of various L-amino acids (**2**) and substituted benzenesulphonyl chloride (**1**) in aqueous medium. Reaction of compounds (**3a-j**) with the appropriate alkyl amine in the presence of catalytic amount of Pd_2_(dba)_3_ afforded the target compounds (**4a-j**, Scheme 2) which were characterized using FTIR, NMR, and HRMS.

### Spectral Characterization

The FTIR spectra of the carboxamides showed a N-H band between 3,489 and 3,160 cm^−1^. The C = O band appeared between 1,733 and 1,603 cm^−1^. These bands indicates successful coupling of the aliphatic amines with the benzenesulphonamides.

The appearance of the peaks between 3.16–3.01, 2.86–2.42, 1.92–1.01, and 0.92–0.64 ppm in the proton NMR is supportive of the formation of the target product.

The carbon-13 NMR showed all the peaks expected of successful coupled products. The C = O peak appeared between 167.86 and 175.22 ppm. All the aromatic and aliphatic peaks were accounted for in the carbon-13 NMR.

The high resolution mass spectrometer (HRMS) peak of the derivatives appeared either as molecular ions (M^+^), M+H^+^, or M–H^−^. The results corresponded to three decimals with the calculated values. The spectra used for the characterization of the new compounds are available as Supporting Materials.

### Molecular Docking

**Figure 1:**
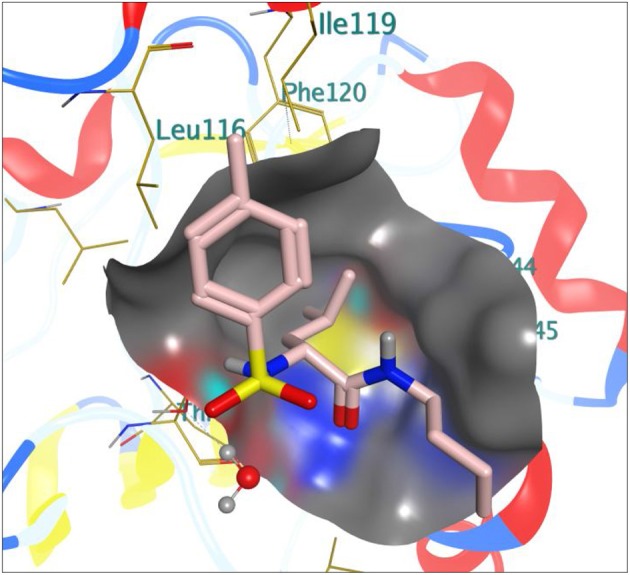
Stereoview of compound **4e** in the binding cavity of 1HD2.

**Figure 2:**
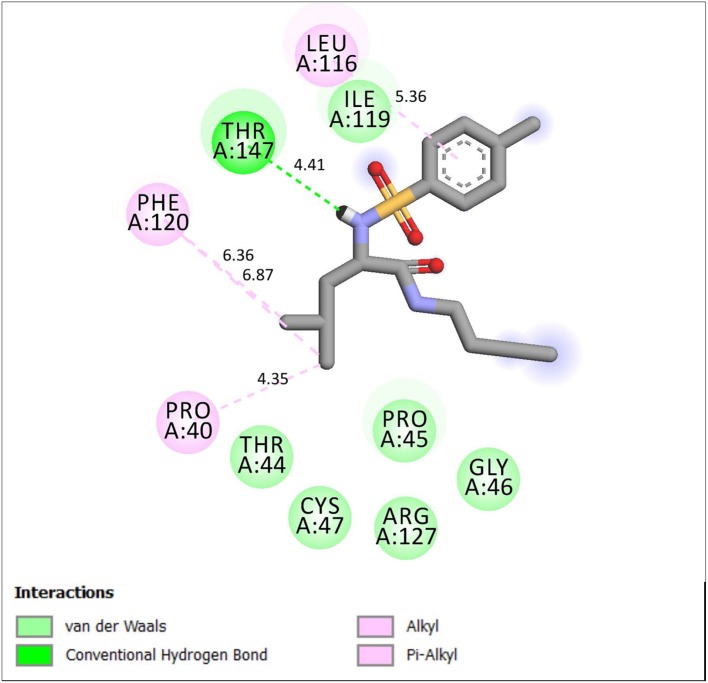
2D representation of binding interaction of compound **4e** and the amino acid residues of 1HD2.

Table 1 shows the binding free energy of our synthesized compounds docked into the binding sites of the receptors: 1HD2, 4WCU, and 2VF5. There were significant binding affinities of the compounds with the receptors when compared to the standard drugs. Compound **4e** showed comparable *in silico* antioxidant activity (−13.05 kcal/mol) as the standard drug (vitamin C) (−13.04 kcal/mol). Likewise, compound **4g** also showed similar *in silico* anti-inflammatory (−11.20 kcal/mol) comparable to indomethacin (−11.38 kcal/mol). However, even though the compounds showed good binding affinity with 2VF5, none showed antibacterial activity that is comparable to ciprofloxacin. We went further to gain insight into the nature of the binding interactions between the compounds and the receptors. Figure 1 shows the stereo view of compound **4e** in the binding cavity of 1HD2, while Figure 2 illustrates how the atoms of compound **4e** interacted with the amino acid residues of 1HD2.

**Table 1:** Binding free energy, ΔG (kcal/mol).

**COMP**	**1HD2** **ΔG (kcal/mol)**	**4WCU** **ΔG (kcal/mol)**	**2VF5** **ΔG (kcal/mol)**
**4a**	−12.03	−10.79	−12.85
**4b**	−10.71	−10.52	−11.35
**4c**	−11.44	−10.15	−10.37
**4d**	−12.60	−10.23	−11.44
**4e**	−13.02	−10.41	−11.71
**4f**	−12.46	−11.03	−12.72
**4g**	−12.83	−11.20	−12.01
**4h**	−12.15	−10.03	−11.96
**4i**	−11.30	−10.21	−11.69
**4j**	−11.54	−10.45	−11.45
Standard drug	−13.04	−11.38	−16.74

*Standard drugs used: antioxidant = Vitamin C; Anti-inflammatory = indomethacin; Antibacteria = ciprofloxacin*.

Compound **4e** is well-fitted in the binding cavity of **1HD2**, sharing the same binding sites with the native ligand. It can be seen from Figure 2 that various chemical interactions played vital roles. There was H-bond interaction between the compound and THR 147 through a H-bond distance of 4.47Å. The π-electrons of the methylphenyl group interacted with LEU 116 through π-alkyl interaction. Other π-alkyl interactions involved PHE 120 and PRO 40. ILE 119, PRO 45, THR 45, and GLY 46 can hydrophobically interact with compound **4e**.

**Figure 3:**
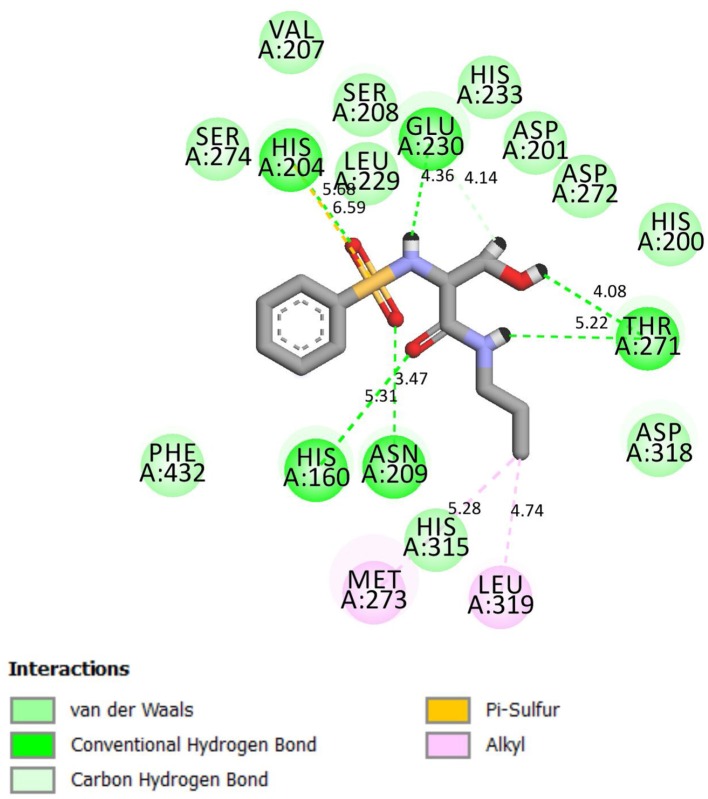
2D representation of binding interaction of compound **4g** and the amino acid residues of 4WCU.

**Figure 4:**
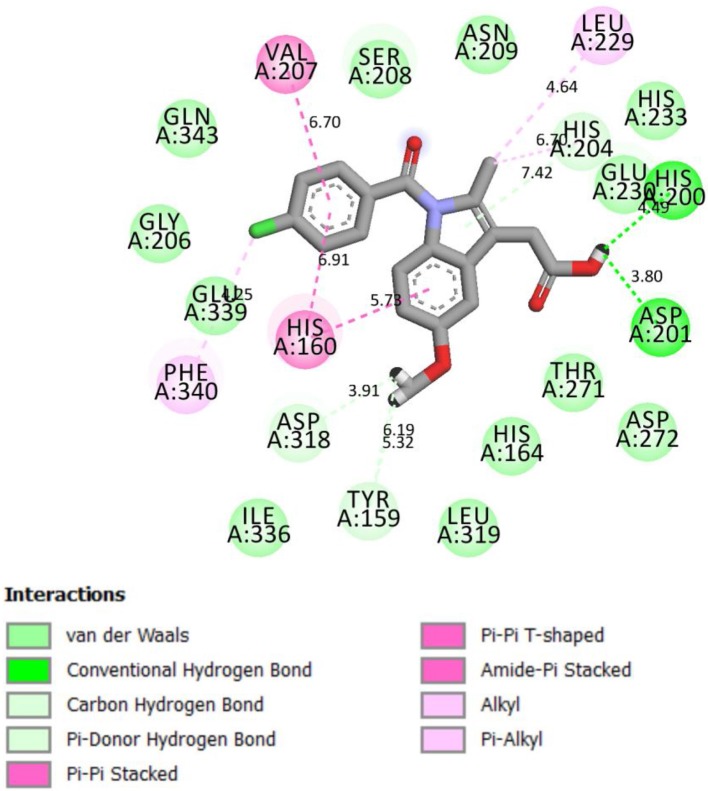
2D representation of binding interaction of indomethacin and the amino acid residues of 4WCU.

Compound **4g** chemically interacted with 4WCU (Figure 3). There were six hydrogen bonds formed in this interaction. The following amino acid residues were involved: HIS 160, ASN 209, THR 271, GLU 230, and HIS 204. π-S interaction was observed the S-atom of **4g** and HIS 204. The details are shown in Figure 3. These interactions were compared with the interactions seen when indomethacin is bound to the binding sites of 4WCU (Figure 4). HIS 200 and ASP 201 formed H-bonds with indomethacin. Details of these interactions are shown in Figure 4. By utilizing different amino acid residues in their interactions, it is possible that compound **4g** and indomethacin may have different mechanisms of reaction in eliciting anti-inflammatory actions.

### Biological Studies

#### *In vivo* Anti-inflammatory Activities

The *in vivo* anti-inflammatory activity showed that all the novel compounds (Table 2) tested had a fascinating inhibition of inflammation (94.69–89.38%) when compared with NSAID indomethacin (78.76%) at 1 h. The most active were compounds **4a** and **4c** with percentage inhibitions of 94.69% each at 1 h. The compounds showed better anti-inflammatory activities at 1 h of the experiment. It was observed that the anti-inflammatory activities decreased with increase in time.

**Table 2:** *In vivo* anti-inflammatory.

**Sample no**	**1 h (%)**	**2 h (%)**	**3 h (%)**
**4a**	94.69	89.66	87.83
**4b**	92.92	87.07	85.22
**4c**	94.69	89.66	87.83
**4d**	92.92	88.79	86.96
**4e**	91.15	83.62	78.26
**4f**	90.45	85,67	83.87
**4g**	93.81	86.21	85.22
**4h**	92.04	86.21	84.35
**4i**	93.81	86.21	84.35
**4j**	89.38	87.07	85.22
Indomethacin	78.76	71.55	66.09

#### *In vitro* Antimicrobial Activities

##### General sensitivity of compounds against microorganism

*Minimum inhibitory concentration (mg/mL).* The antimicrobial studies revealed that most of the novel compounds (MIC 8.90(−6.28 mg/mL) were more potent than the reference drugs (MIC 9.65(−8.39 mg/mL) against the tested microorganisms (Tables 3, 4). Compound **4d** (MIC 6.72 mg/mL) was the most active against *E. coli*. Compound **4h** (MIC 6.63 mg/mL) was the most potent against *S. aureus*. Compound **4a** (MIC 6.67 and 6.45 mg/mL) was the most active against *P. aeruginosa* and *S. typhi*, respectively. Compound **4f** (MIC 6.63 mg/mL) was the most potent against *B. subtilis*. Compounds **4e** and **4h** (MIC 6.63 mg/mL) each were the most active against *C. albicans*, while compound **4e** (MIC 6.28 mg/mL) was the most potent against *A. niger*.

**Table 3:** General sensitivity of compounds against microorganism.

**Sample no**	***E. coli***	***S. aureus***	***P. aeruginosa***	***S. typhi***	***B. subtilis***	***C. albicans***	***A. niger***
**4a**	11	10	8	8	6	4	6
**4b**	12	6	10	-	6	7	7
**4c**	10	9	4	5	4	6	4
**4d**	8	6	-	10	7	-	-
**4e**	8	10	10	8	6	8	8
**4f**	7	4	-	10	8	-	-
**4g**	-	-	3	6	7	5	4
**4h**	11	8	6	6	-	8	8
**4i**	10	-	-	6	4	-	-
**4j**	4	6	6	6	-	-	-
Ciprofloxacin	26	25	25	25	26	-	-
Fluconazole	-	-	-	-	-	24	27

*- = No inhibition*.

**Table 4:** Minimum Inhibitory Concentration (MIC).

**Sample no**	***E. coli***	***S. aureus***	***P. aeruginosa***	***S. typhi***	***B. subtilis***	***C. albicans***	***A. niger***
**4a**	7.44	7.78	6.67	6.45	7.08	-	7.32
**4b**	8.90	6.99	7.70	-	7.08	8.42	8.39
**4c**	7.67	7.89	-	-	-	7.28	-
**4d**	6.72	7.44	-	7.69	8.12	-	-
**4e**	7.01	8.00	7.72	6.60	7.09	6.63	6.28
**4f**	8.39	-	-	7.68	6.63	-	-
**4g**	-	-	-	7.28	8.42	-	-
**4h**	7.64	6.63	7.30	7.51	-	6.63	6.48
**4i**	7.93	-	-	7.43	-	-	-
**4j**	-	7.00	7.30	7.42	-	-	-
Ciprofloxacin	9.65	8.39	9.05	8.68	9.56	-	-
Fluconazole	-	-	-	-	-	9.05	8.39

The structure activity relationship study revealed that the *p*-toluenesulphonamide derivatives possessed better antimicrobial properties than the benzenesulphonamide analogs. This finding underscores the importance of the methyl group at position 4 of the phenyl ring mimicking the structure of *p*-amino benzoic acid which is needed for the synthesis of folic acid by the microorganism.

#### *In vitro* Antioxidant Activities

##### *In vitro* antioxidant activities (% scavenging activity)

*In vitro antioxidant activities (IC_50_).* The *in vivo* antioxidant activities (Tables 5, 6) revealed that all the novel compounds had antioxidant activities, though lower than Vitamin C. Only compound **4e** (IC_50_ 0.3287 mg/mL) had comparable activity with Vitamin C (IC_50_ 0.2090 mg/mL) at 0.25 mg/mL.

**Table 5:** *In vitro* anti-oxidant (% scavenging activity).

**Sample no**	**0.05 mg/mL (%)**	**0.10 mg/mL (%)**	**0.15 mg/mL (%)**	**0.20 mg/mL (%)**	**0.25 mg/mL (%)**
**4a**	2.44	7.07	9.90	14.91	19.15
**4b**	3.34	8.61	13.50	17.74	22.24
**4c**	1.54	6.94	10.28	13.37	23.14
**4d**	2.57	7.46	10.93	15.94	20.18
**4e**	2.83	8.23	13.24	22.62	32.13
**4f**	3.31	7.76	10.58	14.31	18.91
**4g**	2.06	4.76	7.58	11.43	13.88
**4h**	2.19	6.17	9.25	14.91	20.18
**4i**	2.31	6.17	9.90	14.65	21.72
**4j**	3.34	9.00	13.24	19.28	22.75
Vitamin C	11.31	21.85	34.32	48.20	60.15

**Table 6:** *In vitro* antioxidant activities (IC_50_).

**Sample no**	**IC50 (mg/mL)**
**4a**	0.5358
**4b**	0.5080
**4c**	0.3799
**4d**	0.5294
**4e**	0.3287
**4f**	0.4779
**4g**	0.8475
**4hs**	0.5250
**4i**	0.4653
**4j**	0.5826
Vitamin C	0.2090

DPPH assay is used to predict antioxidant activities by mechanism in which the compounds tested inhibit lipid oxidation, so scavenging of DPPH radical and therefore can determine free radical scavenging capacity. The method is used widely due to relatively short time required for the analysis. DPPH free radical is very stable and reacts with compounds that can donate hydrogen atoms. The assay measures the reducing ability of antioxidants toward the DPPH radical.

*Physicochemical properties.* Table 7 shows the physicochemical properties of the synthesized compounds which are useful in the assessment of the drug-likeness.

**Table 7:** Physicochemical properties of the compounds.

**Sample no**	**MilogP**	**TPSA (Å^**2**^)**	**NA**	**MW**	**HBA**	**HBD**	**NV**	**NRB**	**Volume**	**%ABS**
**4a**	2.63	66.48	22	324.45	5	1	0	6	299.56	86.06
**4b**	2.19	66.48	21	310.42	5	1	0	6	283.00	86.06
**4c**	3.64	75.27	23	340.49	5	2	0	9	326.37	83.03
**4d**	3.19	75.27	22	326.46	5	2	0	9	309.81	83.03
**4e**	2.78	75.27	23	358.53	5	2	0	10	327.91	83.03
**4f**	2.33	75.27	22	344.50	5	2	0	10	311.35	83.03
**4g**	3.11	75.27	22	326.46	5	2	0	8	309.57	83.03
**4h**	2.66	75.27	21	312.44	5	2	0	8	293.01	83.03
**4i**	1.35	95.50	21	314.41	6	3	0	8	284.44	76.05
**4j**	0.91	95.50	20	300.38	6	3	0	8	267.88	76.05

*TPSA, total polar surface area; NA, number of atoms; MW, molecular weight; HBA, hydrogen bond acceptor; HBD, hydrogen bond donor; NV, number of violations; NRB, number of rotatable bond*.

Lipinski's rule of five helps to evaluate the bioavailability for oral formulations. An oral drug with a good bioavailability should have MW ≤ 500, HBD ≤ 5, HBA ≤ 10, and Log P(o/w) ≤ 5. A violation of more than one parameter may be an indication of poor bioavailability. Table 7 shows that the synthesized compounds are in agreement with the Lipinski's rule of five. In addition, the TPSA, which is a reflection of the compound's hydrophilicity, is very important in protein-ligand interactions. NRB ≤ 10 and TPSA ≤ 140 Å^2^ would have a high probability of good oral bioavailability in rats. The compounds reported in this research possessed TPSA < 140 and NRB < 10 and as such would not pose oral bioavailability problems if formulated.

## Conclusions

In this paper, we have described an efficient, ecofriendly, and versatile approach to obtain substituted benzenesulphonamides bearing carboxamide. All the compounds were evaluated for their anti-inflammatory, antimicrobial and oxidant activities. Compounds **4a** and **4c** were the most active anti-inflammatory agents, compound **4d** was the most active against *E. coli*, compound **4h** was most active against *S. aureus*, compound **4a** was most active against *P. aeruginosa* and *S. typhi*, compound **4f** was the most active against *B. subtilis*, compounds **4e** and **4h** was the most active against *C. albicans*, compound **4e** was most active against *A. niger*. Compound **4e** had comparable activity to Vitamin C. The derivatives are promising drug candidates which can take care of inflammation and oxidative stress that occurs during microbial invasion while acting as antimicrobial agents.

## Data Availability

All datasets generated for this study are included in the manuscript/Supplementary Files.

## Ethics Statement

The animal study was reviewed and approved by Ethics committee on animal use of the University of Nigeria.

## Author Contributions

All authors listed have made a substantial, direct and intellectual contribution to the work, and approved it for publication.

### Conflict of Interest Statement

The authors declare that the research was conducted in the absence of any commercial or financial relationships that could be construed as a potential conflict of interest.

## References

[B1] Abdel-Aziz H. A. Al-Rashood K. A. ElTahir K. E. H. (2014). Synthesis of *N*-benzenesulphonamid-1*H*-pyrazoles bearing arylsulphonyl moiety: novel celecoxib analogs as potent anti-inflammatory agents. Eur. J. Med. 80, 416–422. 10.1016/j.ejmech.2014.04.065 24794773

[B2] Adeniyi B. A. Odedola H. A. (1996). Antimicrobial potentials of *DiospyrosMespiliforrmis* (*Ebenaceae*). Afr. J. Med. Sci. 255, 211–224. 10457794

[B3] Aissaoui H. Koberstein R. Zumbrunn C. Gatfield J. Brisbare-Roch C. Jenck F. (2008). *N*-glycine-sulphonamides as potent dual Orexin-1/Orexin-2 receptor antagonists. Bioorg. Med. Chem. Lett. 18:5733. 10.1016/j.bmcl.2008.09.079 18845436

[B4] Ali S. A. Qasir A. J. Saor K. Y. (2009). Synthesis of some sulfonamide derivatives with expected antibacterial activity. Iraqi J. Mark. Rec. Cons. Protection 1, 85–93.

[B5] Ananthanarayanan V. S. tetreault S. Saint-Jean A (1993). Interaction of calcium channel antagonists with calcium: spectroscopic and modeling studies on diltiazem and its Ca^2+^ complex. J. Med. Chem. 36:1332. 10.1021/jm00062a004 8496901

[B6] Bhat M. A. Siddiqui N. Khan S. A. (2006). Synthesis of novel sulphonamides as potential antibacterial, antifungal and antimalarial agent. Indian. J. Chem. 54B:134 10.4103/0250-474X.22984

[B7] Bylund J. Burges L. A. Cescutti P. Ernst R. K. Speert D. P. (2006). Exopolysaccharides from *BurkholderiaCenocepacia* inhibit neutrophil chemotaxis and scavenge reactive oxygen species. J. Biol. Chem. 281, 2526–2532. 10.1074/jbc.M510692200 16316987

[B8] Chandak H. S. (2012). Synthesis of isoxazolylbenzenesulphonamide derived from *N*-[4-(2,3-dibromo-2-arylpropanoyl)phenyl]benzenesulphonamide. Der. Pharma. Chem. 4, 1054–1057.

[B9] Christopher W. (2000). Molecular mechanisms that confer antibacterial drug resistance. Nature 406,775–781. 10.1038/35021219 10963607

[B10] Circu M. L. Aw T. Y. (2010). Reactive oxygen species, cellular redox system and apoptosis. Free Radic. Biol. Med. 48, 749–762. 10.1016/j.freeradbiomed.2009.12.022 20045723 PMC2823977

[B11] Declercq J. P. Evrard C. Clippe A. Stricht D. V. Bernard A. Knoops B. (2001). Crystal structure of human peroxiredoxin 5, a novel type of mammalian peroxiredoxin at 1.5 a resolution. J. Mol. Biol. 311:751. 10.1006/jmbi.2001.4853 11518528

[B12] deGasparo M. Whitebread S. (1995). Binding of valsartan to mammalian angio-tensin ATI receptors. Regul. Pept. 59:311 10.1016/0167-0115(95)00085-P 8577935

[B13] Ezeokonkwo M. A. Ogbonna O. N. Okafor S. N. Godwin-Nwakwasi E. U. Ibeanu F. N. Okoro U. C. (2017). Angular phenozaxine ethers as potent multi-microbial targets inhibitors: design, synthesis, and molecular docking studies. Front. Chem. 5:107. 10.3389/fchem.2017.00107 29238706 PMC5712349

[B14] Festus C. Anthony C. E. Ibeji C. U. Okafor S. N. Onwudiwe D. C. Aderoju A. O. (2018). Synthesis, characterization, antimicrobial activity and DFT studies of 2-(pyrimidin-2-ylamino)naphthalene-1,4-dione and its Mn(II), Co(II), Ni(II), and Zn(II) complexes. J. Mol. Struct. 1163, 455–464. 10.1016/j.molstruc.2018.03.025

[B15] Ghorab M. M. Bashandy M. S. Alsaid M. S. (2014). Novel thiophene derivatives with sulphonamides, isoxazole, benzothiazole, quinoline and anthracene moieties as potential anticancer agents. Acta Pharm. 64, 419–431. 10.2478/acph-2014-0035 25531783

[B16] Graul A. Castaner J. (1997). Atovarstatin calcium. Drugs Future 22:968 10.1358/dof.1997.022.09.423212

[B17] Hayley W. Paul W. (2010). Understanding antibiotic resistance. Pharm. J. 274:501 Available online at: https://www.pharmaceutical-journal.com/download

[B18] Hogan B. L. Williams M. Idiculla A. Veysoglu T. Parente E. (2000). Development and validation of a liquid chromatographic method for the determination of the related substances of ramipril in Altace capsules. J. Pharm. Biomed. Anal. 23:651. 10.1016/S0731-7085(00)00342-3 10975240

[B19] Hosseinzadeha N. Serajb S. Bakhshi-dezffolia M. E. Hasanic M. khoshneviszadeha M. Fallah-Bonekohald S. (2013). Synthesis and antidiabetic evaluation of benzenesulfonamide derivatives. Iran. J. Pharm. Res. 12, 325–330. Available online at: http://www.ijpr.ir PMC3813231 24250607

[B20] Islinger M. Grille S. Fahimi H. D. Schrader M. (2012). The peroxisome: an update onmysteries. Histochem. Cell Biol. 137, 547–574. 10.1007/s00418-012-0941-4 22415027

[B21] Jainswal M. Khadikar P. V. Supran C. T. (2004). Topological modeling of lipophilicity diuretic activity and carbonic inhibition activity of benzenesulphonamides: a molecular connectivity approach. Bioorg. Med. Chem. Lett. 14:5666 10.1016/j.bmcl.2004.08.051 15482943

[B22] Knoops B. Goemaere J. Van der Eecken V. Declercq J. P. (2011). Peroxiredoxin 5:structure, mechanism, and function of the mammalian atypical 2-Cys peroxiredoxin. Antioxid. Redox. Signal 15, 817–829. 10.1089/ars.2010.3584 20977338

[B23] Liyana-Pathiranan C. M. Shahidi F. (2005). Antibacterial and antioxidant activities of *AdiantumPedatium* L. J. Phytol. 3, 26–32. 10.1021/jf049320i

[B24] Mahtab R. Srivastava A. Gupta N. Kushwaha S. K. Tripathi A (2014). Synthesis of novel 2-benzylbenzo[d]thiazole-6-sulfonamide derivatives as potential anti-inflammatory agents. J. Chem. Pharm. Sci. 7, 34–38.

[B25] Montalbetti C. A. G. N. Falque V. (2005). Amide bond formation and peptide coupling. Tetrahedron 61:10852 10.1016/j.tet.2005.08.031

[B26] Nakayama T. Sakamoto S. Sassa S. Suzuki S. Kudo H. Nagasawa H. (2005). Paradoxical effect of cytosine arabinoside on mouse leukemia cell line L1210 cells. Anticancer Res. 25:157–160. Available online at: https://pdfs.semanticscholar.org/1be2/8466368a6addffb786142a8c6d7884fda1cc.pdf 15816533

[B27] Papadopoulou V. M. Bloomer D. W. Rosenzweig S. H. Chatelain E. Kaiser M. Wilkinson R. S. (2012). Novel-3-nitro-1-*H*-1,2,4-triazole-based amides and sulphonamides as potential antitrypanosomal agents. J. Med. Chem. 55, 5554–5565. 10.1021/jm300508n 22550999 PMC3375360

[B28] Patchett A. A. (1993). Excursions in drug discovery. J. Med. 36:2051–2058. 10.1021/jm00067a001 8340909

[B29] Prestinaci F. Pezzotti P. Pantosti A. (2015). Antimicrobial resistance: a global multifaceted phenomenon. Pathog. Glob. Health 109, 309–318. 10.1179/2047773215Y.0000000030 26343252 PMC4768623

[B30] Roskoski R. Jr. (2003). Sti-571: an anticancer protein-tyrosine kinase inhibitor. Biochem. Biophys. Res. Commu. 309:717. 10.1016/j.bbrc.2003.08.055 13679030

[B31] Selvam P. Murugesh N. Chandramohan M. Debyser Z. Witvrouw M. (2008). Design, synthesis and anti-HIV activity of novel isatin-sulphonamides. Indian J. Pharm. Sci. 70, 779–782. 10.4103/0250-474X.49121 21369440 PMC3040873

[B32] Shet P. M. Vaidya V. P. Mahadevan K. M. Shivananda M. K. Sreenivasa S. Vijaya-Kumar G. R. (2013). Synthesis, characterization and antimicrobial studies of novel sulphonamides containing substituted naphthofuroyl group. Res. J. Chem. Sci. 3, 15–20. Available online at: www.isca.in

[B33] Siddique M. Saeed A. B. Ahmad S. Dogar N. A. (2013). Synthesis and biological evaluation of hydrazide based sulphonamides. J. Scient. Innovat. Res. 2, 628–634. Available online at: http://www.jsirjounal.com

[B34] Spooner R. Yilmaz O. (2011). The role of Reactive-Oxygen-Specie in microbial persistence and inflammation. Int. J. Mol. Sci. 12, 334–352 10.3390/ijms12010334 21339989 PMC3039955

[B35] Supuran C. T. (2008). Carbonic anhydrase: novel therapeutic applications for inhibitors and activators. Nat. Rev. Drug Disc. 7:168 10.1038/nrd2467 18167490

[B36] Tang G. Lin X. Qiu Z. Li W. Zhu L. Wang L.. (2011). Design and synthesis of benzenesulfonamide derivatives as potent anti-influenza hemagglutinin inhibitors. ACS Med. Chem. Lett. 2, 603–607. 10.1021/ml2000627 24900355 PMC4018120

[B37] Ugwu D. I. Ezema B. E. Eze F. U. Ugwuja D. I. (2014). Synthesis and structural activity relationship study of antitubercular carboxamides. Int. J. Med. Chem. 2014, 1–18. 10.1155/2014/614808 25610646 PMC4295614

[B38] Ugwu D. I. Okoro U. C. Mishra N. K. (2018a). Synthesis, characterization and anthelmintic activity evaluation of pyrimidine derivatives bearing carboxamide and sulphonamide moieties. J. Serbian Chem. Soc. 83, 401–409. 10.2298/JSC170127109U

[B39] Ugwu D. I. Okoro U. C. Mishra N. K. (2018b). Synthesis, characterization and *in vivo* antitrypanosomal activities of new carboxamides bearing quinoline moiety. PLoS ONE 13:e0191234 10.1371/journal.pone.0191234 29324817 PMC5764481

[B40] Ugwu D. I. Okoro U. C. Ukoha P. O. Okafor S. Ibezim A. Kumar N. M. (2017). Synthesis, characterization, molecular docking and *in vivo* antimalarial properties of new carboxamides bearing sulphonamide. Eur. J. Med. Chem. 135, 349–369. 10.1016/j.ejmech.2017.04.029 28460310

[B41] Zhang K. Y. Ibrahim P. N. Gillete S. Gideon B. (2005). Phosphodiesterase-4 as a potential drug target. Expert Opin. Ther. Targets 9, 1283–1305. 10.1517/14728222.9.6.1283 16300476

