# Supplementary material for: Biological Activity Evaluation of Some New Benzenesulphonamide Derivatives
Source: Front Chem. 2019 Sep 18;7:634. doi: 10.3389/fchem.2019.00634 (PMC6759663; doi:10.3389/fchem.2019.00634)
Supplement: Supplementary file 1 [file Data_Sheet_1.PDF]

Supporting document

***N*-butyl-1-[(4-methylphenyl)sulphonyl]pyrolidine-2-carboxamide (4a)**

Yield (0.22 g, 66.7 %), Mp,128-130 ºC. UV (λmax): 213.00 nm (ε=427.5 m^2/mol). FTIR (KBr, cm^{-1}): 3160 (NH), 2959 (C-H aromatic), 2871, 2795 (C-H aliphatic), 1629 (C=O), 1569, 1450, (C=C), 1342, 1394 (2S=O), 1200, 1156 (SO_2NH), 1092, 1055 (C-N).^{1}HNMR (DMSOd$_6$,400 MHz)δ: 7.68-7.64 (m, 2H, ArH), 7.35-7.33 (m, 2H, ArH), 3.82 (m, 1H, CH-C=O), 3.22 (m, 1H, CHa of CH_2-N), 3.09-3.07 (m, 1H, CHb of CH_2-N), 2.67 (m, 2H, CH_2-NH), 2.24 (s, 3H, CH_3-Ar), 1.69 (m, 2H,CH_2CHC=O), 1.47 (m, 2H, CH_2-CH_2-N), 1.28-1.26 (m, 4H, 2CH2, CH_2CH_2-CH_3), 0.85-0.80 (m, 3H,CH_3-CH_2).^{13}CNMR (DMSOd$_6$, 400MHz)δ: 175.22 (C=O), 143.17,136.28, 130.07, 127.63, (four aromatic carbons), 63.71, 48.75, 38.99, 31.18, 30.29, 24.66, 21.50, 19.80,14.10, (nine aliphatic carbons).HRMS (m/z): 325.1588 (M+H), calculated, 325.1586.

***N*-butyl-1-(phenylsulphonyl)pyrrolidine-2-carboxamide (4b)**

Yield (0.27 g, 87.1 %), Mp, 114-116 ºC. UV (λmax): 202.00 nm(ε=398.3 m^2/mol).FTIR (KBr, cm^{-1}): 3489 (NH), 2960 (C-H aromatic), 2874, 2796 (C-H aliphatic), 1629 (C=O), 1569, 1446 (C=C), 1394, 1334 (2S=O), 1193, 1156 (SO_2NH), 1088, 1014 (C-N).^{1}HNMR (DMSOd$_6$,400 MHz)δ: 7.80-7.78 (d, J=8.24 Hz, 2H, ArH), 7.62-7.52 (m, 3H, ArH), 3.88-3.86 (t, J=3.42 Hz, 1H, CH-C=O),3.14-3.11 (t, J=6.88 Hz, 2H, CH_2-N), 2.68-2.65 (m, 2H, CH_2-NH), 1.70 (m, 2H, CH_2-CH-C=O), 1.51-1.43 (m, 2H, CH_2-CH_2-N), 1.29-1.24 (m, 4H, 2CH_2, CH_2-CH_2-CH_3), 0.84-0.81 (m, 3H,CH_3-CH_2).^{13}CNMR (DMSOd$_6$, 400MHz)δ: 174.99 (C=O), 139.20,132.97,129.61, 127.56, (four aromatic carbons), 63.74, 48.72, 31.18, 30.38, 24.67, 19.79, 14.11, (seven aliphatic carbons).HRMS (m/z): 311.1430 (M+H), calculated, 311.1429.

***N*-butyl-4-methyl-2-(4-methylbenzenesulphonamido)pentanamide (4c)**

Yield (0.31 g, 88.6 %), Mp, 110-112 ºC. UV (λmax): 202.00 nm(ε=445.2 m^2/mol). FTIR (KBr, cm^{-1}): 3250 (NH), 3049 (C-H aromatic), 2960, 2866, 2751 (C-H aliphatic), 1640 (C=O), 1580, 1461 (C=C), 1394, 1342 (2S=O), 1286, 1159 (SO_2NH), 1099, 977 (C-N).^{1}HNMR (DMSOd$_6$,400 MHz)δ: 8.18-8.16 (d, J=8.40 Hz, 1H, NH), 7.86-7.80 (m, 2H, Ar), 6.72-6.70 (m, 2H, ArH), 3.36-3.35 (t, J=4.40 Hz, 1H, CH-C=O),3.16-3.14 (m, 2H, CH_2-NH), 3.06-3,01 (m, 6H,3CH_2), 2.46 (s, 3H, CH_3-Ar), 1.14-1.10 (m, 9H, 3CH_3). ^{13}CNMR (DMSOd$_6$, 400MHz)δ: 167.86 (C=O), 154.70, 137.58,131.70,127.16, 125.18, 123.91 (six aromatic carbons), 64.26, 52.65, 46.07, 39.32, 36.45, 8.93 (six aliphatic carbons).HRMS (m/z): 340.1822 (M^+), calculated, 340.1821.

***N*-butyl-4-methyl-2-[(phenylsulphonyl)amino]pentanamide (4d)**

Yield (0.25 g, 78.1 %), Mp, 116-118 ºC. UV (λmax): 202.00 nm(ε=422.1 m^2/mol).FTIR (KBr, cm^{-1}): 3258 (NH), 3064 (C-H aromatic), 2960, 2870, (C-H aliphatic), 1640 (C=O), 1573, 1428 (C=C), 1387, 1338 (2S=O), 1290, 1152 (SO_2NH), 1014, 973 (C-N).^{1}HNMR (DMSOd$_6$,400

You created this PDF from an application that is not licensed to print to novaPDF printer (http://www.novapdf.com)

MHz)δ: 7.73-7.71 (d, J=7.80 Hz, 2H, ArH), 7.56-7.47 (m, 3H, ArH), 3.19 (m, 1H, CH-C=O), 2.61-2.57 (m, 2H, CH_2-NH), 1.72-1.65 (m, 1H, CH-$(CH_3)_2$), 1.44-1.38 (m, 2H, CH_2-CHC=O), 1.31-1.18 (m, 4H, CH_2), 0.91-0.87 (m, 3H, CH_3), 0.82-0.72 (m, 6H, 2(CH_3)). ^{13}CNMR (DMSOd_6, 400MHz)δ: 174.30 (C=O), 141.38, 132.58,129.37,127.13 (four aromatic carbons), 56.48, 43.28, 29.77, 24.51,23.47, 22.61, 19.72, 14.04 (eight aliphatic carbons).HRMS (m/z): 327.1744 (M+H), calculated, 327.1742.

_N_-butyl-2-{[(4-methylphenyl)sulphonyl]amino}-4-(methylsulphanyl)butanamide (4e)

Yield (0.36 g, 100 **%**), Mp, 124-126 °C.UV (λmax): 202.00 nm (ε=399.4 m^2/mol). FTIR (KBr, cm^{-1}): 3250 (NH), 3049 (C-H aromatic), 2960, 2922, 2870 (C-H aliphatic), 2624 (S-CH_3), 1640 (C=O), 1580, 1521, 1435, 1402 (C=C), 1361, 1316 (2S=O), 1208, 1152 (SO_2NH), 1088, 1047 (C-N).^{1}HNMR (DMSOd_6,400 MHz)δ: 7.62-7.60 (d, J=7.32 Hz, 2H, ArH), 7.32-7.30 (d, J=7.92 Hz, 2H, ArH), 3.17-3.14 (t, J=5.18 Hz, 1H, CH-C=O), 2.67-2.64 (m, 2H, CH_2-NH), 2.39-2.32 (m, 3H, CH_3-Ar), 2.25 (s, 3H, CH_3-S), 1.99-1.92 (t, J=5.64 Hz, 2H, CH_2-S), 1.78-1.76 (m, 2H, CH_2-CH), 1.46-1.39 (m, 2H, CH_2-CH_2-NH), 1.29-1.20 (m, 2H, CH_2-CH_3), 0.87-0.84 (t, J=5.20 Hz, 3H, CH_3-CH_2). ^{13}CNMR (DMSOd_6, 400MHz)δ: 172.08 (C=O), 143.00, 137.84,130.00,127.28 (four aromatic carbons), 56.68, 41.18, 38.90, 33.54,29.88, 21.48, 19.67, 15.03, 14.04, (nine aliphatic carbons).HRMS (m/z) : 357.1310 (M-H), calculated, 357.1307.

_N_-butyl-2-(4-methylphenylsulphonyl)-2-[(phenylsulphonyl)amino]butanmide (4f)

Yield (0.30 g,92 **%**), Mp, 120-121 °C.UV (λmax): 202.00 nm(ε=406.9 m^2/mol).FTIR (KBr,cm^{-1}): 3246 (NH),3067 (CH aromatic), 2917, 2918 (CH aliphatic), 2657 (-S-CH_3), 1710 (C=O), 1584,1416 (C=C aromatic),1326, 1233 (2S=O), 1155, 1088, 969, 928 (C-N).^{1}HNMR (DMSOd_6,400 MHz)δ: 7.88-7.87 (d, J=7.40 Hz, 2H, ArH), 7.65-7.64 (t, J=7.32 Hz, 3H, ArH), 3.15-3.13 (t, J=6.22 Hz, 1H, CH-C=O), 2.66-2.64 (m, 2H, CH_2-NH), 2.23 (s, 3H, CH_3-S), 1.97-1.95 (m, 2H, CH_2-S), 1.79-1.77 (m, 2H, CH_2-CH), 1.42-1.38 (m, 2H, CH_2-CH_2-NH), 1.27-1.24 (m, 2H, CH_2-CH_3), 0.84-0.82 (t, J=5.20 Hz, 3H, CH_3-CH_2). ^{13}CNMR (DMSOd_6, 400MHz)δ: 173.38 (C=O), 143.46, 138.65,130.50,127.28 (four aromatic carbons), 56.68, 41.18, 38.90, 33.54,29.88, 19.67, 15.03, 14.04, (eight aliphatic carbons).HRMS (m/z): 344.1229 (M^+), calculated 344.1228.

_N_-butyl-3-hydroxy-2-{[(4-methyl)sulphonyl]amino}butanamide (4g)

Yield (0.28 g, 84.8 %), Mp, 162-164 °C.UV (λmax): 213.00 nm (ε=421.7 m^2/mol). FTIR (KBr, cm^{-1}): 3399 (OH), 3250 (NH), 2959 (C-H aromatic), 2934, 2874, (C-H aliphatic), 1603 (C=O), 1513, 1465 (C=C), 1379, 1327 (2S=O), 1159, 1122 (SO_2NH), 1092, 1036 (C-N, C-O).^{1}HNMR (DMSOd_6,400 MHz)δ:7.69- 7.57 (m, 2H, ArH), 7.48-7.46 (m, 2H, ArH), 3.77-3.76 (d, J=6,32, Hz, 1H, CH-C=O), 3.08-3.06 (m, 1H, CH-OH), 2.56-2.53 (m, 2H, CH_2-NH), 1.41-1.39 (m, 2H, CH_2-CH_2-NH), 1.33-1.31 (m, 2H, CH_2-CH_3), 0.98-0.96 (d, J=5.96 Hz, 3H, CH_3-CHOH), 0.83-0.81 (m, 3H, CH_3-CH_2). ^{13}CNMR (DMSOd_6, 400MHz)δ: 172.64 (C=O), 142.44,

You created this PDF from an application that is not licensed to print to novaPDF printer (http://www.novapdf.com)

132.94,128.71,127.84 (four aromatic carbons), 68.01, 60.14, 38.95, 29.70, 21.56,19.64, 19.09, 14.02, (eight aliphatic carbons).HRMS (m/z): 327.1380 (M-H), calculated, 328.1379.

***N*-butyl-3-hydroxy-2-[(phenylsulphonyl)amino]butanamide (4h)**

Yield (0.29 g,90.6 %), Mp, 140-142 °C.UV (λmax): 213.00 nm (ε=362.1 m^2/mol).FTIR (KBr, cm^{-1}): 3401 (OH), 3191 (NH), 3056 (C-H aromatic), 2960, 2933, 2870 (C-H aliphatic), 1733 (C=O), 1595, 1528, 1476 (C=C), 1387, 1305 (2S=O), 1238, 1141 (SO_2NH), 1077, 973 (C-N, C-O).^{1}HNMR (DMSOd_6,400 MHz)δ: 7.75 (m, 2H, ArH), 7.59-7.51 (m, 3H, ArH), 3.70-3.69 (d, J=4.56 Hz, 1H, CH-C=O), 3.082 (m, 1H, CH-OH), 2.67-2.65 (m, 2H, CH_2-NH), 1.43-1.41 (m, 2H, CH_2-CH_2-NH), 1.28-1.22 (m, 2H, CH_2-CH_3), 0.92-0.89 (d, J=5.96 Hz, 3H, CH_3-CHOH), 0.84-0.79 (m, 3H, CH3-CH2). ^{13}CNMR (DMSOd_6, 400MHz)δ: 171.60 (C=O), 140.44, 132.94,129.61,127.24 (four aromatic carbons), 68.01, 60.14, 38.95, 29.70,19.64, 19.09, 14.02, (seven aliphatic carbons).HRMS (m/z): 315.1379 (M+H), calculated, 315.1378.

***N*-butyl-3-hydroxy-2-{[(4-methylphenyl)sulphonyl]amino}propanamide (4i)**

Yield (0.29 g,90.6 %), Mp=134-136 °C. UV (λmax): 203.00 nm (ε=330.9 m^2/mol). FTIR (KBr, cm^{-1}): 3466 (OH), 3243 (NH), 3060 (C-H aromatic), 2960, 2933, 2873 (C-H aliphatic), 1733 (C=O), 1595, 1494, 1461 (C=C), 1365, 1324 (2S=O), 1163, 1122 (SO_2NH), 1092, 1033 (C-N, C-O).^{1}HNMR (DMSOd_6,400 MHz)δ: 7.89-7.87 (d, J=7.36 Hz,1H, NH), 7.62-7.61 (d, J=6.44 Hz, 2H, ArH), 7.32-7.30 (d, J=6.44 Hz, 2H, ArH), 3.51-3.48 (m, 1H, CH), 3.30 (s-br, 1H,OH), 2.47-2.46 (m, 2H, CH_2-NH), 2.33(s, 3H, CH_3-Ar), 1.62-1.58 (m, 2H, CH_2-OH), 1.34-1.29 (m, 2H, CH_2-CH_2-NH), 1.09-1.01 (m, 2H, CH_2-CH_3) 0.76-0.71 (t, J=5.48 Hz, 3H, CH_3-CH_2).^{13}CNMR (DMSOd_6, 400MHz)δ: 172.36 (C=O), 149.93, 147.24,128.72,124.78 (four aromatic carbons), 60.89, 37.32, 24.85, 21. 44, 19.54, 15.96, 11.46 (seven aliphatic carbons).HRMS (m/z): 314.1303 (M^+), calculated, 314.1300.

***N*-butyl-3-hydroxy-2-[(phenylsulphonyl)amino]propanamide (4j)**

Yield (0.28 g,93.3 %), Mp=120-122 °C.UV (λmax): 203.00 nm(ε=364.7 m^2/mol). FTIR (KBr, cm^{-1}): 3391 (OH), 3243 (NH), 2963 (C-H aromatic), 2875 (C-H aliphatic), 1733 (C=O), 1599, 1446 (C=C), 1387, 1320 (2S=O), 1252, 1160 (SO_2NH), 1092, 1026 (C-N, C-O).^{1}HNMR (DMSOd_6,400 MHz)δ: 7.94-7.93 (d, J=7.36 Hz,1H, NH), 7.63-7.62 (d, J=6.44 Hz, 2H, ArH), 7.31-7.29 (d, J=6.40 Hz, 3H, ArH), 3.53-3.49 (m, 1H, CH), 3.30 (s-br, 1H,OH), 2.44-2.42 (m, 2H, CH_2-NH), 1.58-1.56 (m, 2H, CH_2-OH), 1.44-1.42 (m, 2H, CH_2-CH_2-NH), 1.06-1.04 (m, 2H, CH_2-CH_3) 0.66-0.64 (t, J=6.32 Hz, 3H, CH_3-CH_2).^{13}CNMR (DMSOd_6, 400MHz)δ: 170.76 (C=O), 148.43, 146.64,127.74,124.78 (four aromatic carbons), 63.44, 38.44, 37.32, 24.85,15.96, 11.46 (six aliphatic carbons).HRMS (m/z): 299.1069 (M-H), calculated, 299.1066.

***N*-butyl-1-[(4-methylphenyl)sulphonyl]pyrrolidine-2-carboxamide**

You created this PDF from an application that is not licensed to print to novaPDF printer (http://www.novapdf.com)

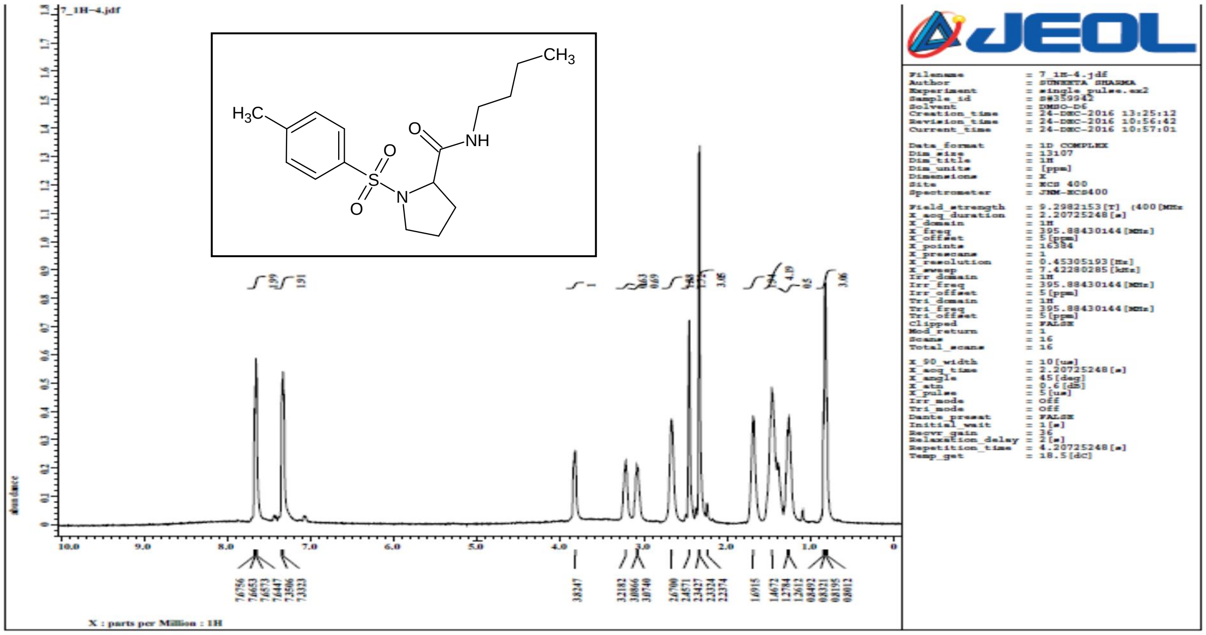

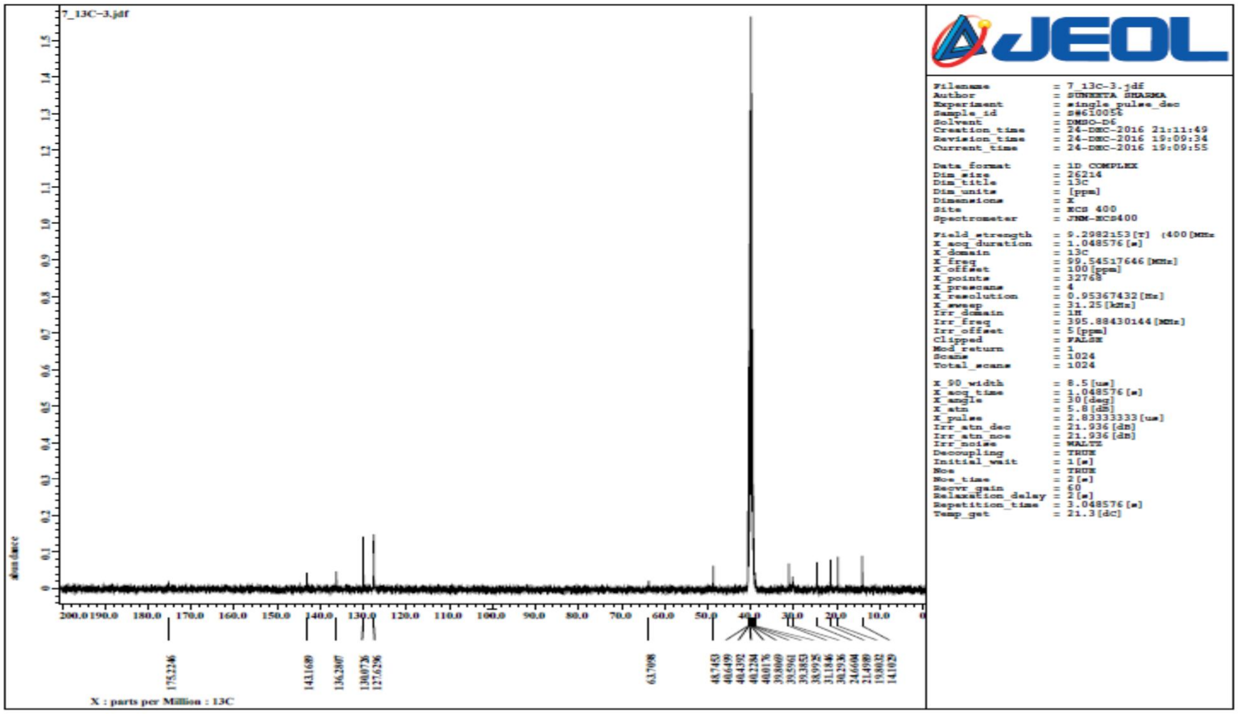

You created this PDF from an application that is not licensed to print to novaPDF printer (http://www.novapdf.com)

Agilent Technologies

Sample ID:7
Sample Scans:16
Background Scans:16
Resolution:8
System Status:Good
File Location:C:\Program Files\Agilent\MicroLab PC\Results\7_2016-12-21T16-13-19.a2r

Method Name:Transmittance Method
User:Admin
Date/Time:2016-12-21T16:13:19.506+01:00
Range:4000 - 650
Apodization:Happ-Genzel

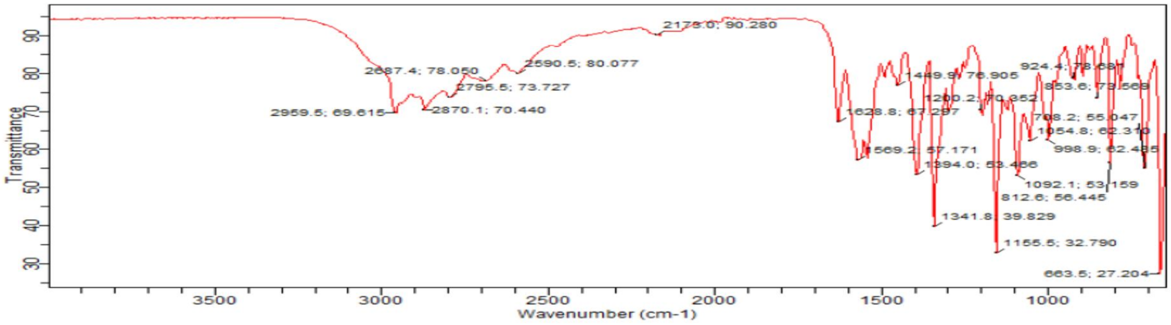

12/22/2016 10:23:33 AM Page 1 of 1

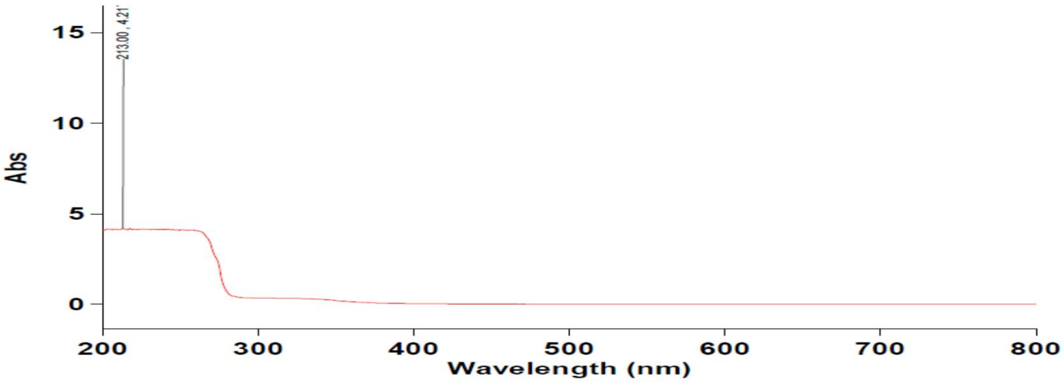

Scan Analysis Report

Report Time : Thu 22 Dec 10:19:50 AM 2016
Method:
Batch:
Software version: 4.20(468)
Operator:

Zero Report

Read	Abs(800.00)
Zero	0.0515

Sample Name: sample7

Collection Time 12/22/2016 10:21:32 AM

Peak Table
Peak Style Maximum Peak
Peak Threshold 0.0100
Range 800.00nm to 200.00nm

Wavelength (nm)	Abs
213.00	4.217

You created this PDF from an application that is not licensed to print to novaPDF printer (http://www.novapdf.com)

***N*-butyl-1-(phenylsulphonyl)pyrrolidine-2-carboxamide**

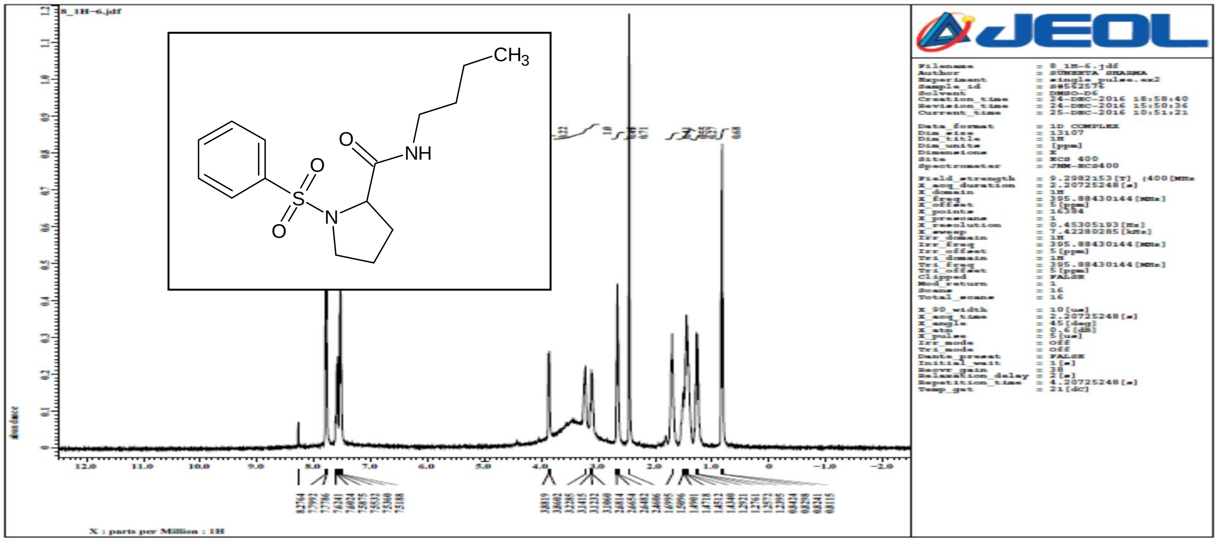

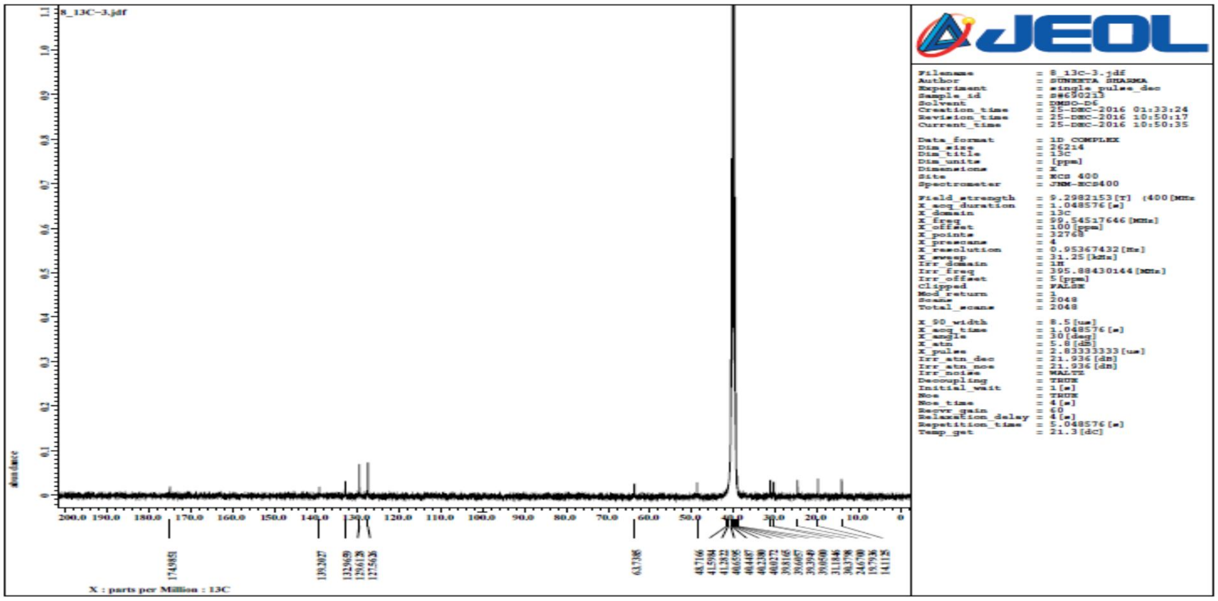

You created this PDF from an application that is not licensed to print to novaPDF printer (http://www.novapdf.com)

Agilent Technologies

Sample ID:8	Method Name:Transmittance Method
Sample Scans:16	User:Admin
Background Scans:16	Date/Time:2016-12-21T16:17:32.601+01:00
Resolution:8	Range:4000 - 650
System Status:Good	Apodization:Happ-Genzel

File Location:C:\Program Files\Agilent\MicroLab PC\Results\8_2016-12-21T16-17-32.a2r

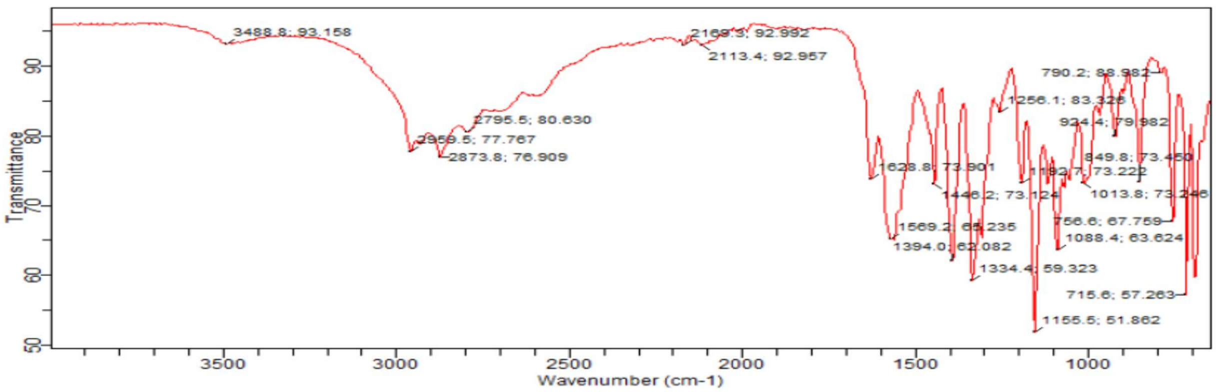

12/22/2016 10:26:30 AM   Page 1 of 1

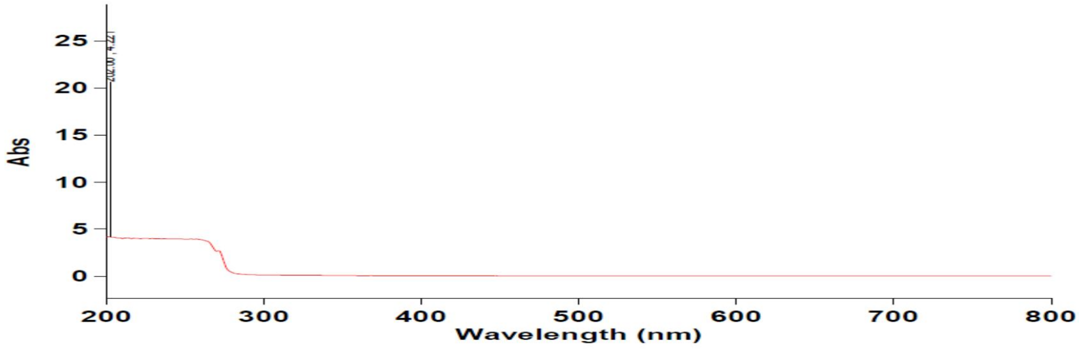

Scan Analysis Report

Report Time : Thu 22 Dec 10:24:32 AM 2016
Method:
Batch:
Software version: 4.20(468)
Operator:

Zero Report

Read	Abs(800.00)
Zero	0.0515

Sample Name: sample8

Collection Time   12/22/2016 10:24:39 AM

Peak Table	
Peak Style	Maximum Peak
Peak Threshold	0.0100
Range	800.00nm to 200.00nm

Wavelength (nm)	Abs
202.00	4.221

You created this PDF from an application that is not licensed to print to novaPDF printer (http://www.novapdf.com)

N-butyl-4-methyl-2-(4-methylbenzenesulphonamido)pentanamide

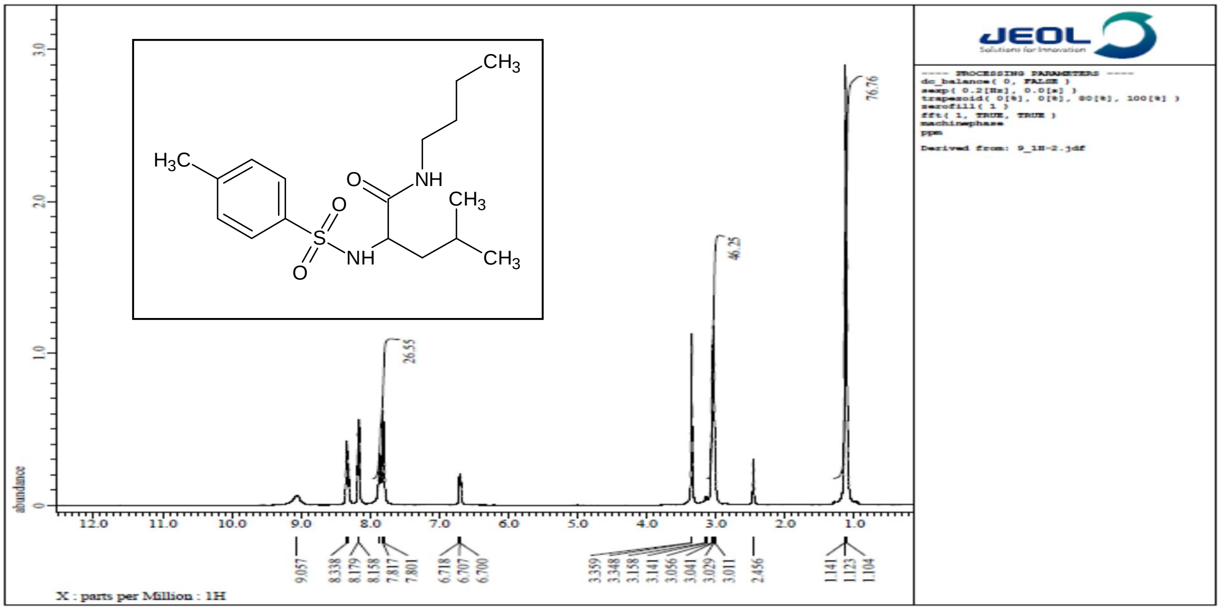

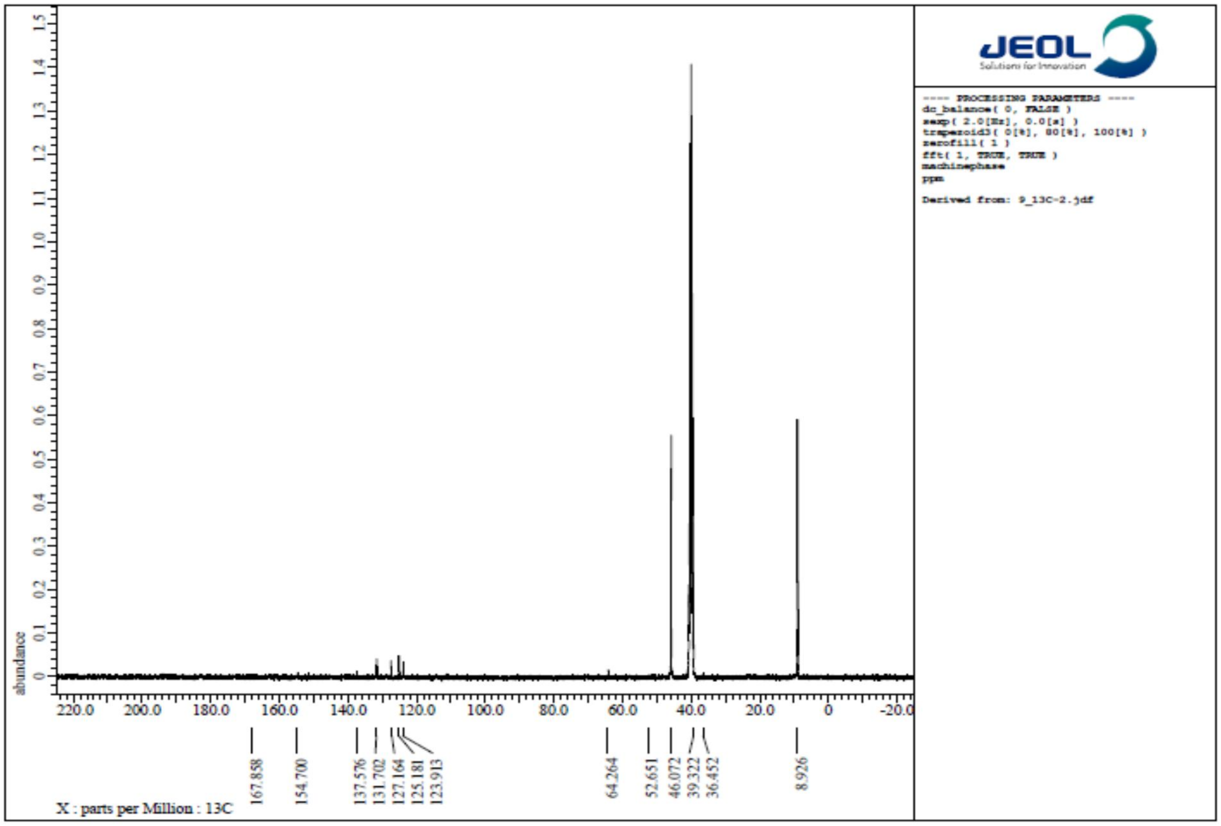

You created this PDF from an application that is not licensed to print to novaPDF printer (http://www.novapdf.com)

Agilent Technologies

Sample ID:9
Sample Scans:16
Background Scans:16
Resolution:8
System Status:Good
File Location:C:\Program Files\Agilent\MicroLab PC\Results\9_2016-12-21T16-06-09.a2r

Method Name:Transmittance Method
User:Admin
Date/Time:2016-12-21T16:06:09.569+01:00
Range:4000 - 650
Apodization:Happ-Genzel

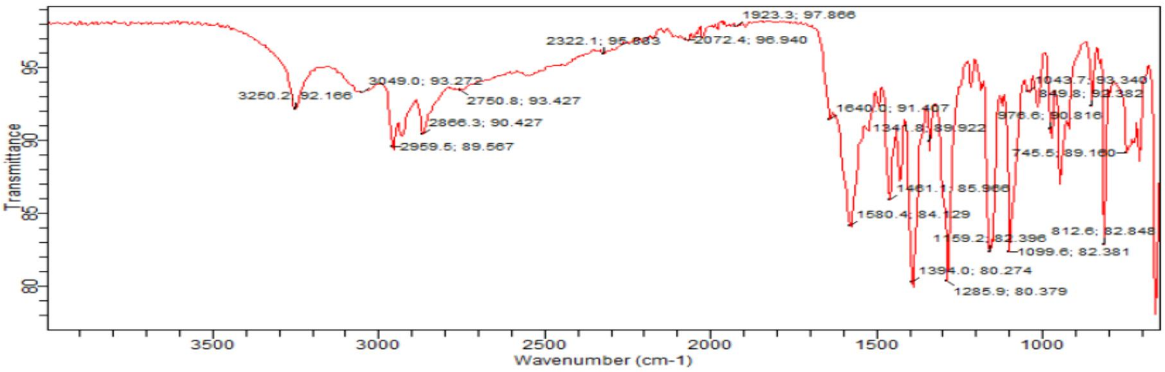

12/22/2016 10:41:49 AM Page 1 of 1

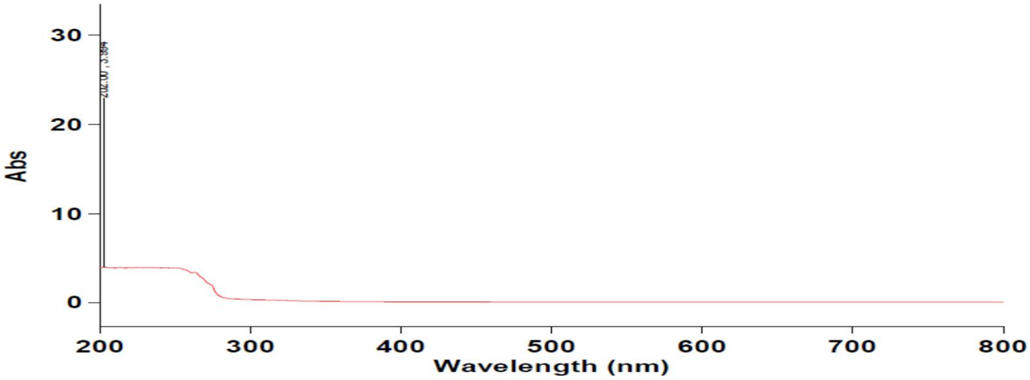

Scan Analysis Report

Report Time : Thu 22 Dec 10:28:05 AM 2016
Method:
Batch:
Software version: 4.20(468)
Operator:

Zero Report

Read	Abs(800.00)
Zero	0.0515

Sample Name: sample9

Collection Time 12/22/2016 10:28:11 AM

Peak Table
Peak Style Maximum Peak
Peak Threshold 0.0100
Range 800.00nm to 200.00nm

Wavelength (nm)	Abs
202.00	3.994

You created this PDF from an application that is not licensed to print to novaPDF printer (http://www.novapdf.com)

N-butyl-4-methyl-2-[(phenylsulphonyl)amino]pentanamide

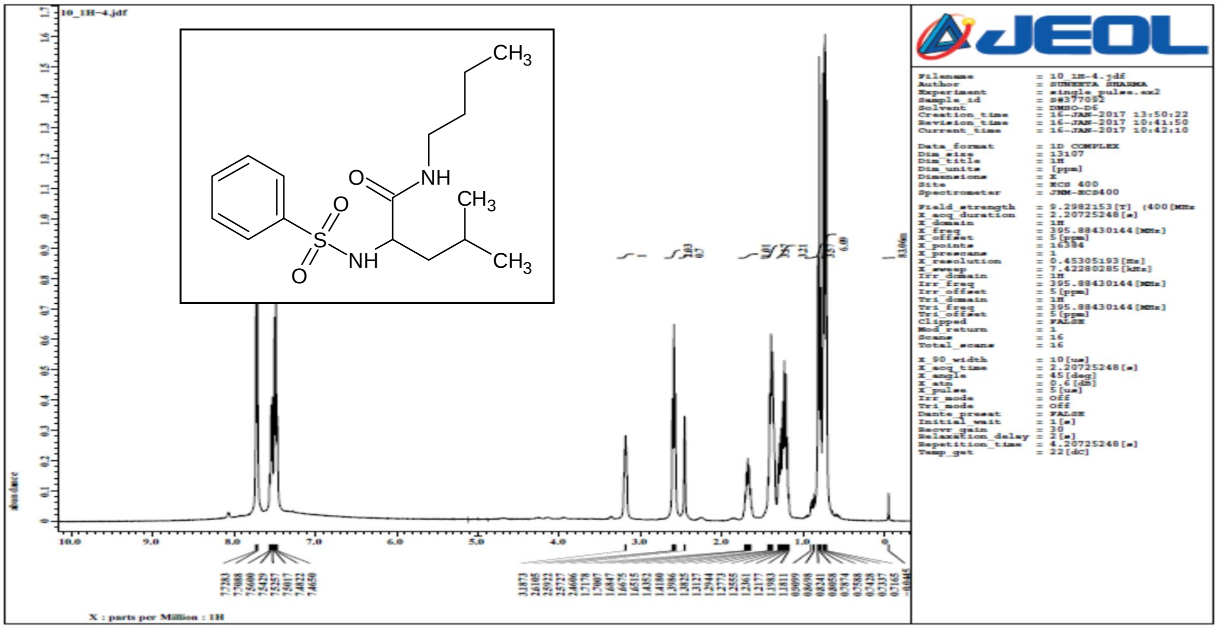

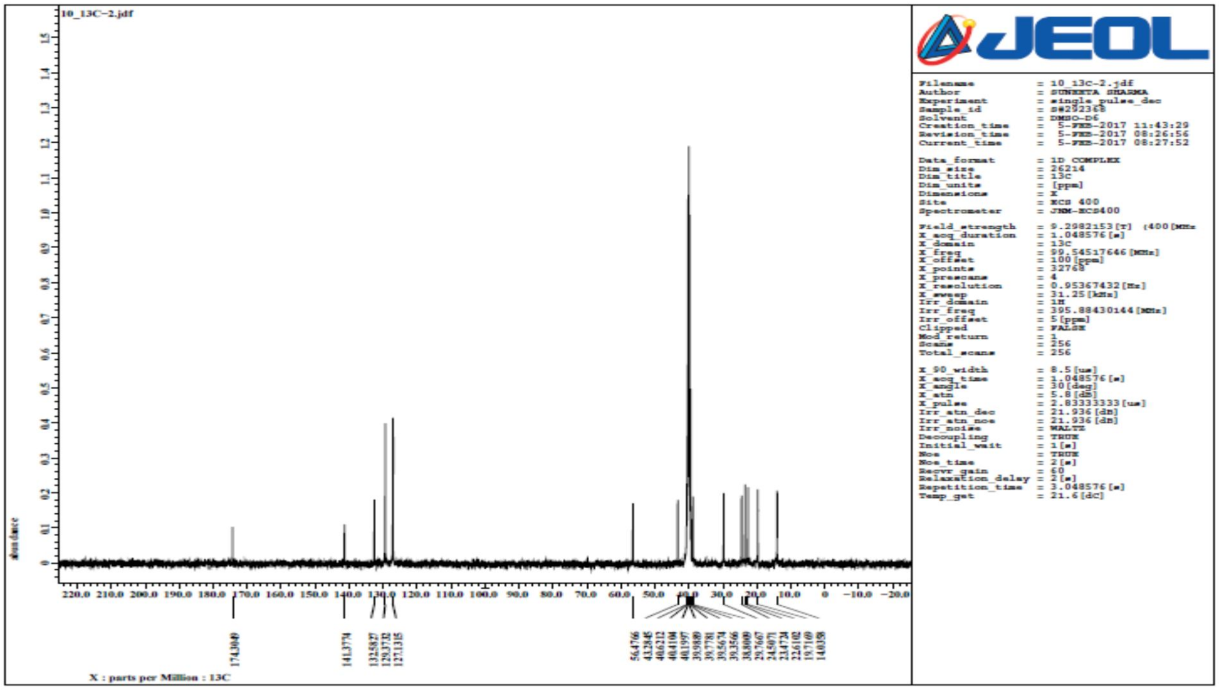

You created this PDF from an application that is not licensed to print to novaPDF printer (http://www.novapdf.com)

Agilent Technologies

Sample ID:10
Sample Scans:16
Background Scans:16
Resolution:8
System Status:Good
Method Name:Transmittance Method
User:Admin
Date/Time:2016-12-21T16:29:33.432+01:00
Range:4000 - 650
Apodization:Happ-Genzel
File Location:C:\Program Files\Agilent\MicroLab PC\Results\10_2016-12-21T16-29-33.a2r

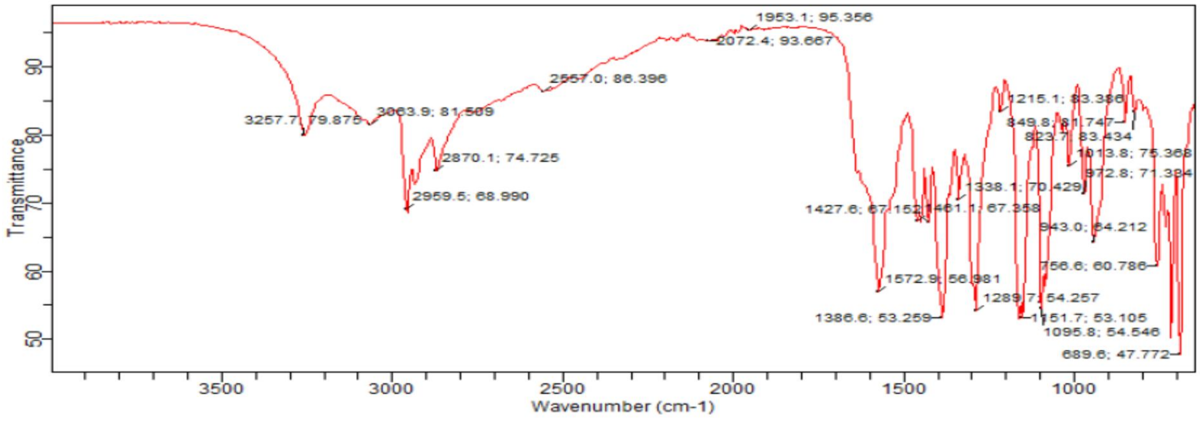

12/22/2016 10:48:12 AM Page 1 of 1

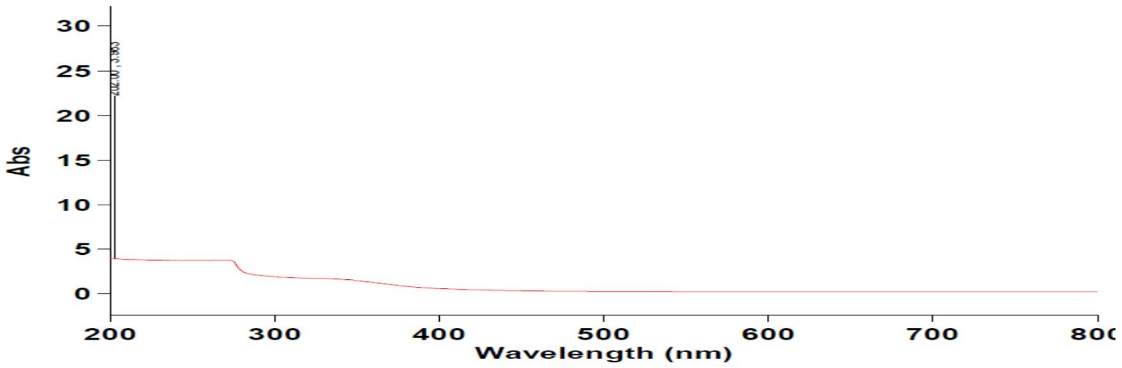

Scan Analysis Report

Report Time : Thu 22 Dec 10:44:13 AM 2016
Method:
Batch:
Software version: 4.20(468)
Operator:

Zero Report

Read	Abs(800.00)
Zero	0.0515

Sample Name: sample10

Collection Time 12/22/2016 10:44:20 AM

Peak Table
Peak Style: Maximum Peak
Peak Threshold: 0.0100
Range: 800.00nm to 200.00nm

Wavelength (nm)	Abs
202.00	3.983

You created this PDF from an application that is not licensed to print to novaPDF printer (http://www.novapdf.com)

N-butyl-2-{[(4-methylphenyl)sulphonyl]amino}-4-(methylsulphanyl)butanamide

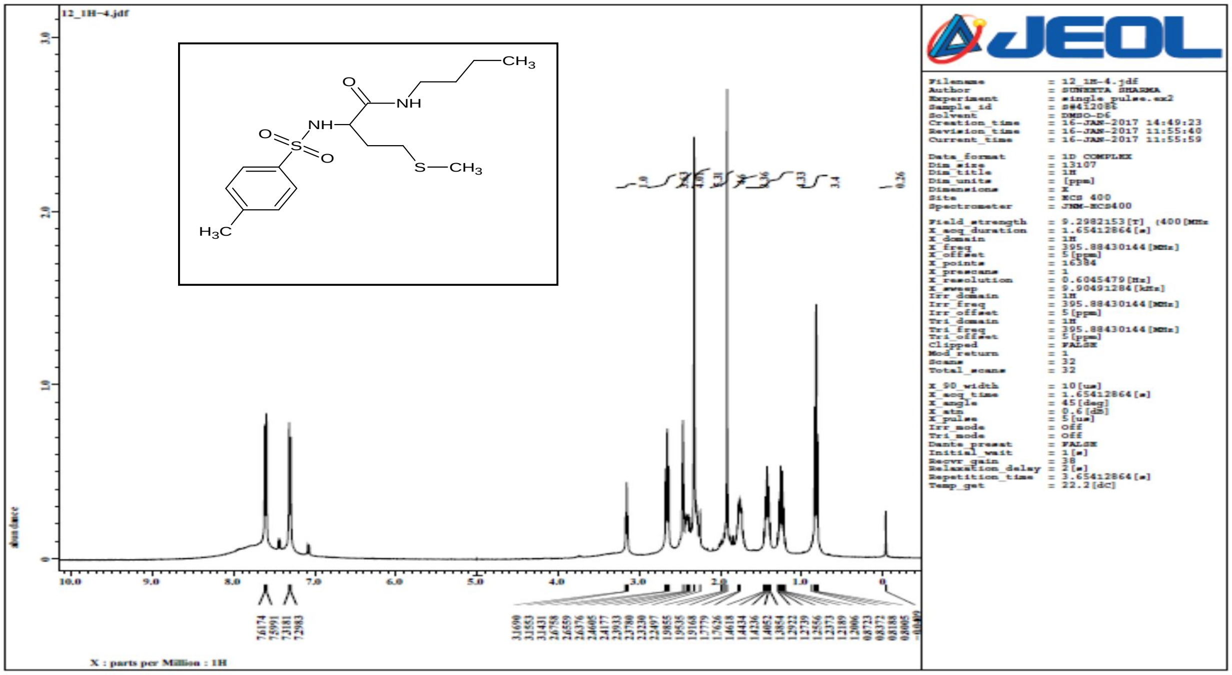

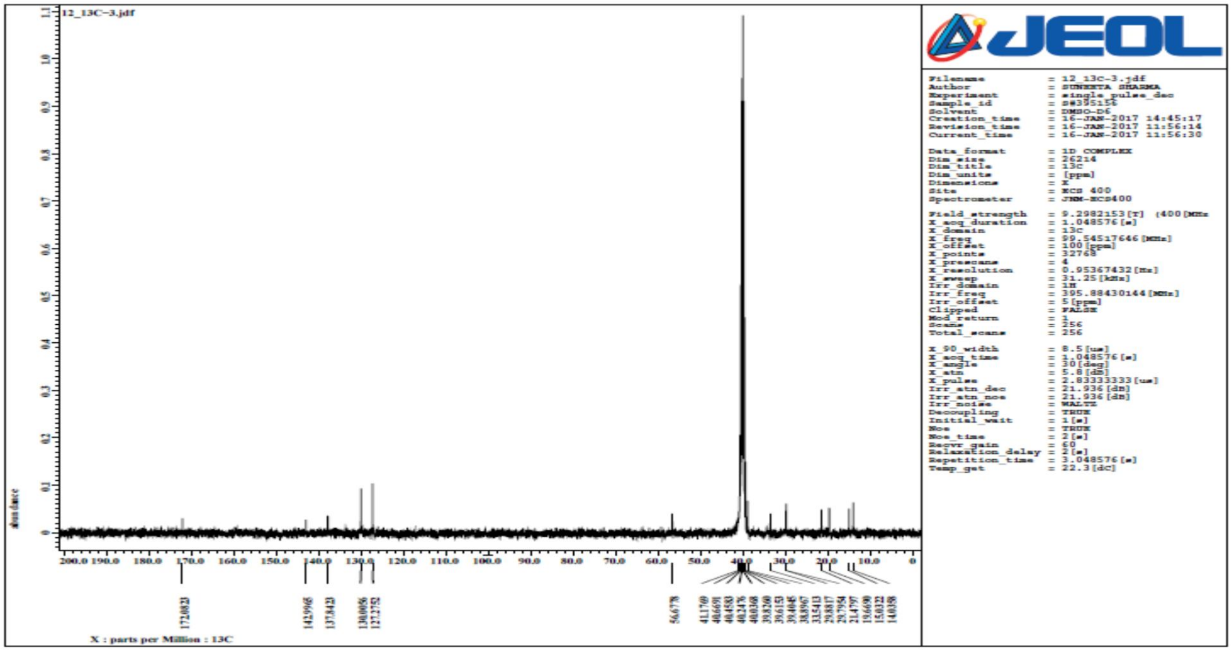

You created this PDF from an application that is not licensed to print to novaPDF printer (http://www.novapdf.com)

Agilent Technologies

Sample ID:12
Sample Scans:16
Background Scans:16
Resolution:8
System Status:Good
File Location:C:\Program Files\Agilent\MicroLab PC\Results\12_2016-12-21T16-14-45.a2r

Method Name:Transmittance Method
User:Admin
Date/Time:2016-12-21T16:14:45.213+01:00
Range:4000 - 650
Apodization:Happ-Genzel

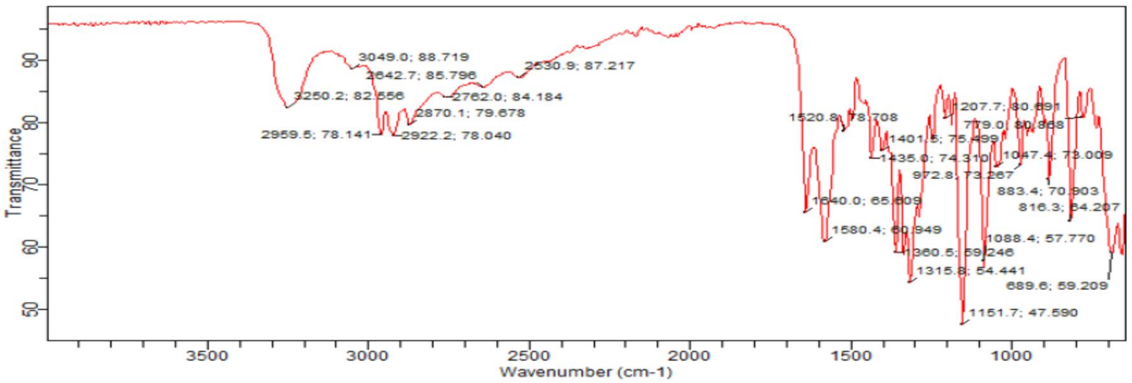

12/22/2016 11:01:14 AM Page 1 of 1

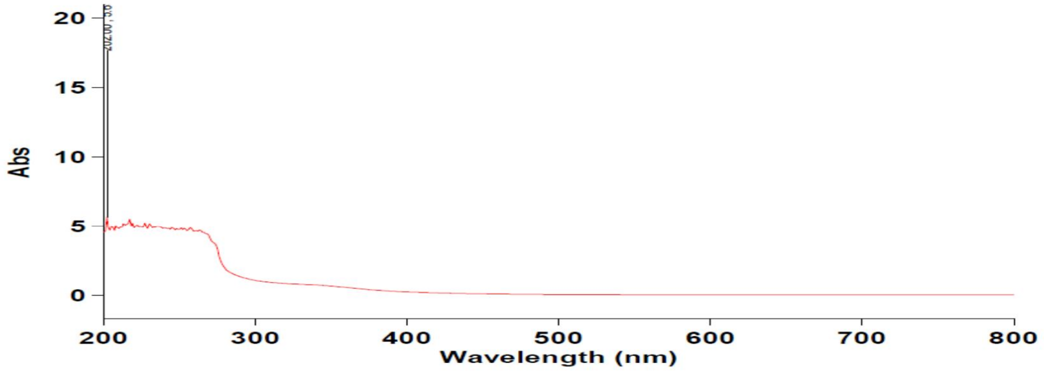

Scan Analysis Report

Report Time : Thu 22 Dec 10:59:11 AM 2016
Method:
Batch:
Software version: 4.20(468)
Operator:

Zero Report

Read	Abs(800.00)
Zero	0.0515

Sample Name: sample12

Collection Time 12/22/2016 10:59:17 AM

Peak Table
Peak Style Maximum Peak
Peak Threshold 0.0100
Range 800.00nm to 200.00nm

Wavelength (nm)	Abs
202.00	5.692

You created this PDF from an application that is not licensed to print to novaPDF printer (http://www.novapdf.com)

N-butyl-3-hydroxy-2-[(phenylsulphonyl)amino]butanamide

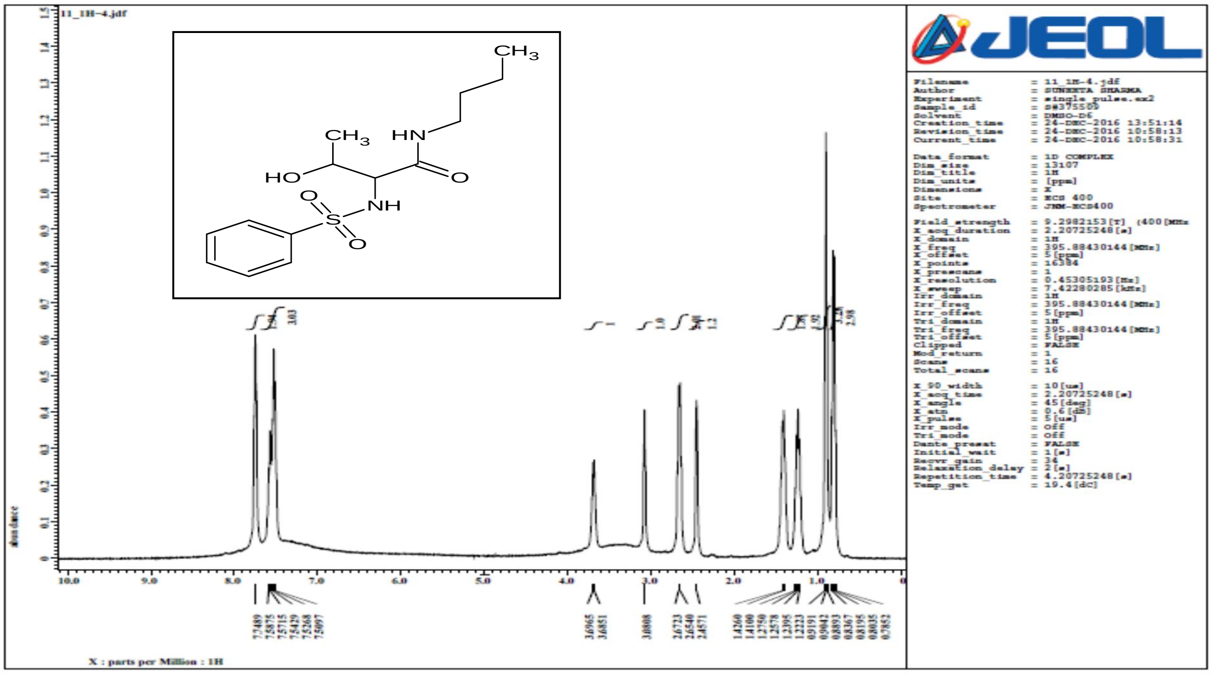

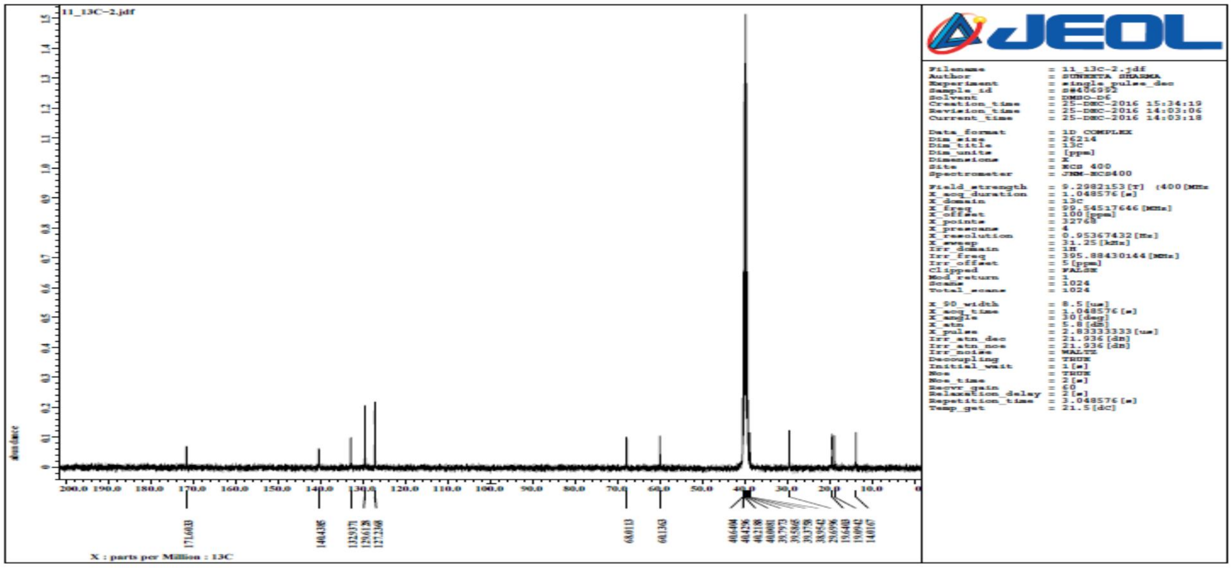

You created this PDF from an application that is not licensed to print to novaPDF printer (http://www.novapdf.com)

N-butyl-3-hydroxy-2-{[(4-methylphenyl)sulphonyl]amino}propanamide

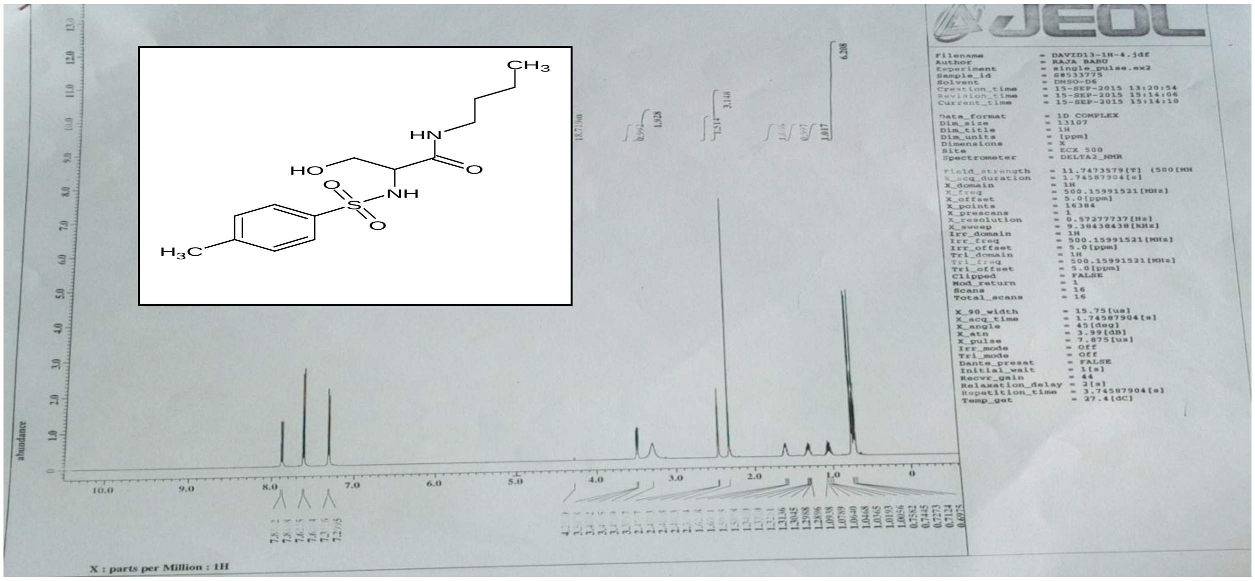

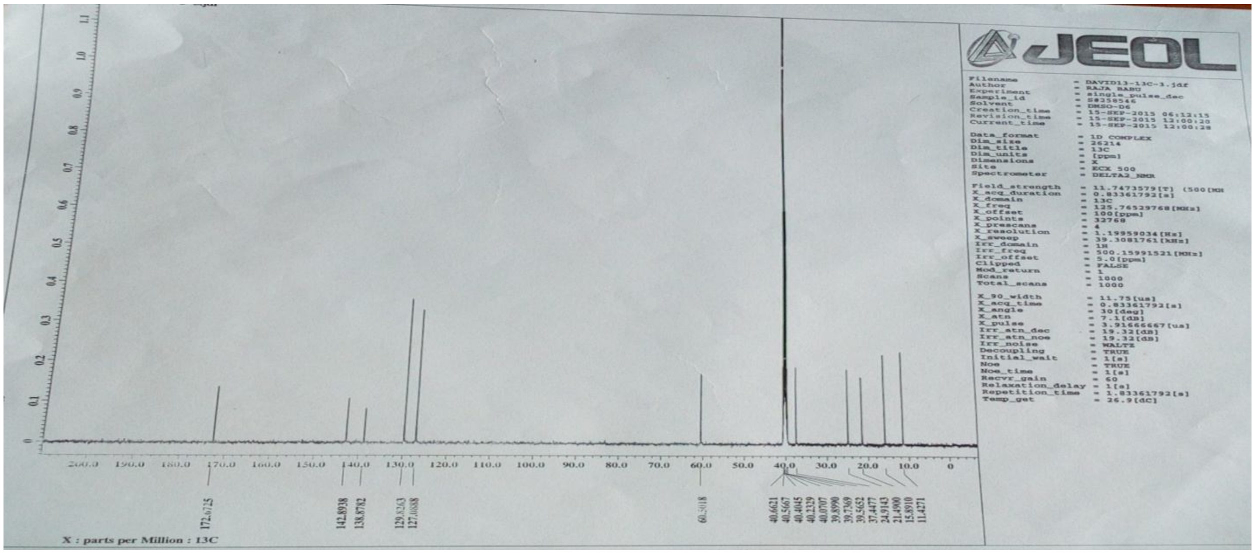

You created this PDF from an application that is not licensed to print to novaPDF printer (http://www.novapdf.com)

Agilent Technologies

Sample ID:24	Method Name:Transmittance Method
Sample Scans:16	User:Admin
Background Scans:16	Date/Time:2016-12-21T16:04:48.169+01:00
Resolution:8	Range:4000 - 650
System Status:Good	Apodization:Happ-Genzel

File Location:C:\Program Files\Agilent\MicroLab PC\Results\24_2016-12-21T16-04-48.a2r

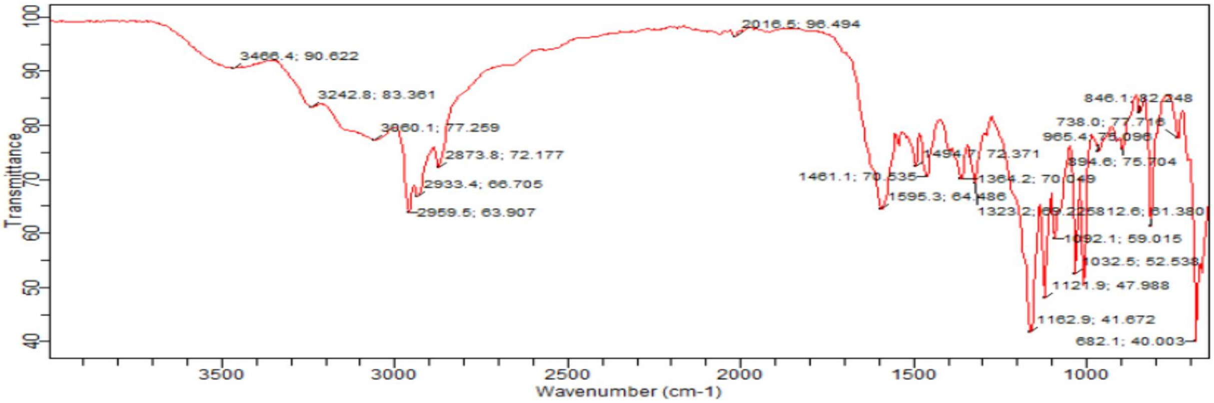

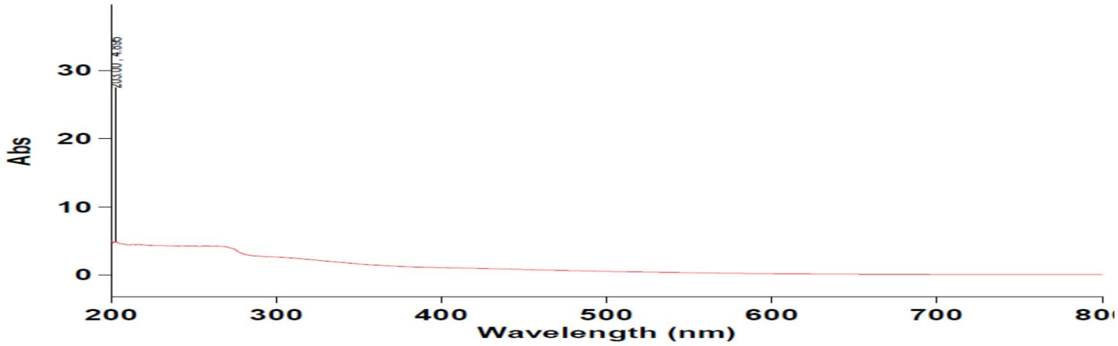

Scan Analysis Report

Report Time : Thu 22 Dec 12:02:36 PM 2016
Method:
Batch:
Software version: 4.20(468)
Operator:

Zero Report

Read	Abs(800.00)
Zero	0.0515

Sample Name: sample24

Collection Time 12/22/2016 12:02:42 PM

Peak Table	
Peak Style	Maximum Peak
Peak Threshold	0.0100
Range	800.00nm to 200.00nm

Wavelength (nm)	Abs
203.00	4.895

You created this PDF from an application that is not licensed to print to novaPDF printer (http://www.novapdf.com)
